# ‘Multi-Epitope-Targeted’ Immune-Specific Therapy for a Multiple Sclerosis-Like Disease via Engineered Multi-Epitope Protein Is Superior to Peptides

**DOI:** 10.1371/journal.pone.0027860

**Published:** 2011-11-29

**Authors:** Nathali Kaushansky, Nicole Kerlero de Rosbo, Rina Zilkha-Falb, Reut Yosef-Hemo, Lydia Cohen, Avraham Ben-Nun

**Affiliations:** Department of Immunology, The Weizmann Institute of Science, Rehovot, Israel; Washington University, United States of America

## Abstract

Antigen-induced peripheral tolerance is potentially one of the most efficient and specific therapeutic approaches for autoimmune diseases. Although highly effective in animal models, antigen-based strategies have not yet been translated into practicable human therapy, and several clinical trials using a single antigen or peptidic-epitope in multiple sclerosis (MS) yielded disappointing results. In these clinical trials, however, the apparent complexity and dynamics of the pathogenic autoimmunity associated with MS, which result from the multiplicity of potential target antigens and “epitope spread”, have not been sufficiently considered. Thus, targeting pathogenic T-cells reactive against a single antigen/epitope is unlikely to be sufficient; to be effective, immunospecific therapy to MS should logically neutralize concomitantly T-cells reactive against as many major target antigens/epitopes as possible. We investigated such “multi-epitope-targeting” approach in murine experimental autoimmune encephalomyelitis (EAE) associated with a single (“classical”) or multiple (“complex”) anti-myelin autoreactivities, using cocktail of different encephalitogenic peptides vis-a-vis artificial multi-epitope-protein (designated Y-MSPc) encompassing rationally selected MS-relevant epitopes of five major myelin antigens, as “multi-epitope-targeting” agents. Y-MSPc was superior to peptide(s) in concomitantly downregulating pathogenic T-cells reactive against multiple myelin antigens/epitopes, via inducing more effective, longer lasting peripheral regulatory mechanisms (cytokine shift, anergy, and Foxp3+ CTLA4+ regulatory T-cells). Y-MSPc was also consistently more effective than the disease-inducing single peptide or peptide cocktail, not only in suppressing the development of “classical” or “complex EAE” or ameliorating ongoing disease, but most importantly, in reversing chronic EAE. Overall, our data emphasize that a “multi-epitope-targeting” strategy is required for effective immune-specific therapy of organ-specific autoimmune diseases associated with complex and dynamic pathogenic autoimmunity, such as MS; our data further demonstrate that the “multi-epitope-targeting” approach to therapy is optimized through specifically designed multi-epitope-proteins, rather than myelin peptide cocktails, as “multi-epitope-targeting” agents. Such artificial multi-epitope proteins can be tailored to other organ-specific autoimmune diseases.

## Introduction

Multiple sclerosis (MS) is an inflammatory disease of the CNS characterized by neurological impairment of variable extent, resulting from primary demyelination and axonal damage. Although the etiology of the disease is not yet known, ample evidence suggests that autoimmune mechanisms directed against myelin components in the CNS play an important pathogenic role [1], [2]. It is widely accepted that the development/progression of MS results from a non-physiological activation of potentially encephalitogenic T cells reactive against CNS components, as evident from studies showing that transferred activated line T-cells or clones reactive against a CNS myelin antigen are sufficient to initiate a full-blown clinical experimental autoimmune encephalomyelitis (EAE) in naïve syngeneic recipients [3], [4], [5], Accordingly, among the numerous approaches that have been proposed for immunotherapy of MS, the immune-specific approach, which can specifically neutralize the pathogenic myelin-reactive T cells, while leaving the innocent immune cells intact, is the ultimate goal in disease therapy.

Several approaches have been proposed for immune-specific treatment of MS as a prototypic organ-specific autoimmune disease [2], [6]. These include administration of a disease-specific protein/peptide in a soluble form [7], [8], [9] or as a DNA vaccine [10], [11], autologous T-cell vaccination [3], [12], anti-clonotypic TCR antibodies [13], [14], immunization with TCR peptide [15], [16], or administration of an MS-related monomeric MHC/peptide polypeptide [17], [18]. However, although effective in animal models, none of these approaches have been translated into a practicable therapy for human MS. Among these approaches, the antigen-based strategies have long been proposed as a powerful means for induction of antigen-specific peripheral tolerance and treatment of autoimmune diseases, as systemic administration of antigen/peptide was shown to be specific and highly effective in neutralizing pathogenic antigen-specific autoimmune T-cells in laboratory animals [19], [20]. Yet, clinical trials with injections of soluble myelin basic protein (MBP) [21], [22], native MBP peptide [23], or MBP altered peptide ligand (MBP-APL) [24], [25], for the treatment of MS have yielded disappointing results. These clinical trials, however, were designed to target pathogenic T-cells reactive against only a single target antigen/epitope, without sufficient consideration of the apparent complexity and dynamics of the pathogenic autoimmunity associated with MS due to multiplicity of target antigens and the emerging “epitope spread” [26].

Indeed, myelin basic protein (MBP), proteolipid protein (PLP), and myelin oligodendrocyte glycoprotein (MOG) are well-recognized target antigens in MS [1], [27]. More recently, also the myelin-associated oligodendrocytic basic protein (MOBP) and oligodendrocyte-specific protein (OSP) have been recognized as additional important target antigens for the potentially pathogenic myelin-reactive T cells associated with MS [27]. This multiplicity of potential target antigens suggests that in different MS patients, the primary pathogenic anti-myelin autoimmunity initiating the disease might be directed against different myelin target proteins. Moreover, the neo-autoreactivities that can emerge as a result of inter- and intra-molecular “epitope spread” [26], [28], as demonstrated during the course of chronic EAE [29], [30] and suggested to be associated with disease progression in MS [31], [32], further contribute to the complexity and the dynamics of the pathogenic anti-myelin autoimmunity associated with MS. Such complex and dynamic autoimmunity imposes major difficulties in devising antigen-based immune-specific therapy of the disease, as by the time of the definite disease diagnosis, the primary pathogenic autoreactive T cells in a given patient may have already shifted or expanded to reactivity against several myelin antigens. Hence, since the specificity of the primary pathogenic T cells may not only be different in different patients, but also can shift or expand in the same patient with the progression of the disease, targeting pathogenic autoreactivity directed against a single myelin target antigen/epitope is unlikely to be a sufficiently effective therapy for MS. Therefore, a conceivably more effective approach to immune-specific therapy of MS would be if potentially pathogenic T-cells reactive against all known major target myelin antigen(s)/epitope(s) would be concomitantly neutralized, regardless of against which of the target myelin antigen(s)/epitope(s) the pathogenic T cells are specifically directed in each given patient upon disease onset, or at any given time of disease progression.

We have previously demonstrated the feasibility of such a concomitant multi-targeting approach using an artificial protein encompassing limited MS/EAE-related epitopes of MBP, PLP, and MOG, as a multi-epitope targeting agent [33]. This artificial protein was highly effective in concomitantly downregulating T-cells reactive against PLP and MOG peptide epitopes [33]. We have now generated a more comprehensive and human-specific (HLA-DR2 relevant) multi-targeting agent, designated Y-MSPc (Y-MS related Protein), the product of a synthetic gene encoding a wide spectrum of rationally selected MS-relevant epitopes of all the five known major encephalitogenic target antigens in MS; MBP, PLP, MOG, MOBP, and OSP. In this study, the specifically designed artificial multi-epitope protein (Y-MSPc) and a cocktail of MS-relevant myelin peptides, as strategies for concomitant multi-epitope targeting, were investigated and compared for their efficacy in: 1) concomitant downregulation of multiple pathogenic T-cells reactive against different myelin antigens; 2) induction of peripheral regulatory mechanisms; and, 3) in the suppression as well as in the treatment of EAE associated with pathogenic anti-myelin autoreactivities against a single (“classical” EAE) or multiple (“complex EAE”) myelin antigens. The results strongly suggest that antigen-based immune-specific therapy of organ-specific autoimmune diseases associated with complex autoimmunity, such as MS, by a specifically designed artificial multi-epitope protein is superior to therapy by single or a cocktail of disease-relevant peptides.

## Materials and Methods

### Mice

Female C57Bl/6J and SJL/J mice were purchased from Jackson Laboratories (Bar Harbor, ME, USA) or obtained from the Weizmann Institute colony. (C57Bl/6JxSJL/J)F1 (BSF1) mice were bred at the Weizmann Institute Animal Facility. C57Bl.FoxP3GFP mice were a kind gift from Dr. Kuchroo, V. J. [34]. (C57Bl.FoxP3GFPxSJL/J)F1 mice were bred at the Weizmann Institute Animal Facility. All mice were 2–3 month-old when used in the experiments. The IACUC of the Weizmann Institute has approved the experiments, permit number: 03530710-3, which were performed in accordance to its relevant guidelines and regulations.

### Myelin antigens, peptides, and the Y-MSPc multi epitope protein

**Figure 1:**
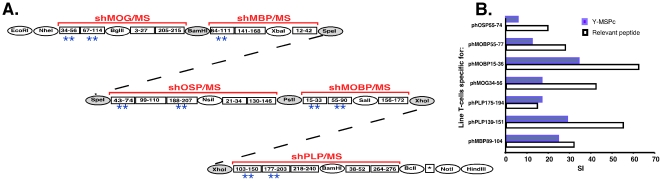
Generation of Y-MSPc and its immunofunctional properties. (A) *Scheme for the construction of the Y-MSPc-encoding gene*. **, denotes epitope clusters containing human sequences that are encephalitogenic for C57Bl/6J (H-2^b^) [36], [39], [55], [56] or SJL/J (H-2^s^) [57], [58], [59], [60], [61], [62] mice and also for BSF1 (H-2^b/s^) mice (Table S1). DNA and derived amino acid sequences of Y-MSPc are shown in Fig. S1. (B) *Y-MSPc is adequately processed and presented to relevant epitope-specific encephalitogenic T-cell lines*. Epitope-specific line T cells (1.5×10^4^/well) raised from peptide-primed LN cells isolated from BSF1 mice were tested for their proliferative response to their priming peptide (5 µg/ml ) or Y-MSPc (5 µg/ml ), in the presence of syngeneic irradiated APCs (5×10^5^/well). Each bar on the histograms represents the mean stimulation index (SI) of triplicate cultures (SD<10%). Background cpm ± SD for phMBP89-104-, phPLP139-151-, phPLP175-194, phMOG34-56, phMOBP15-36-, phMOBP55-77-, and phOSP55-74-specific line T-cells were 249±15, 374±21, 1555±216, 1767±436, 2937±121, 261±69, and 3217±426, respectively.

Recombinant human MOG (rhMOG) was prepared as described previously [35]. Mouse spinal cord homogenate (MSCH) was prepared as described previously [4]. The myelin peptides used in this study, listed in Table S2 (over 80% purity), were synthesized in the laboratory of Prof. M. Fridkin, Department of Organic Chemistry, The Weizmann Institute of Science. Y-MSPc (Y-MS-relevant multi-epitope Protein; the Y is an arbitrary symbol for the series of multi-epitope proteins designed for several organ-specific autoimmune diseases in our laboratory) is a recombinant artificial protein encompassing multiple human myelin epitopes as depicted in Fig. 1A. The Y-MSPc was generated as described in detail in Text S1 (S1.3.). The DNA and deduced amino acid sequences, and the expression of Y-MSPc gene in E. coli and the isolation of the Y-MSPc protein are also shown and detailed in Text S1 (S1.3.).

### T-cell lines and T-cell proliferative responses

Antigen-specific T-cell lines were selected in vitro as described previously [4] from LN cells of mice that had been primed 9 days before with antigen (100 µg myelin peptide) emulsified in CFA containing 150 µg Mycobacterium tuberculosis H37Ra (Cat. No: 3114-25, Difco Laboratories, Detroit, MI). All T-cell lines were maintained in vitro in medium containing IL-2 with alternate stimulation with the antigen, every 10–14 days as previously described [4]. Proliferation assays of T-cell lines were performed as previously described [4], [36].

### Induction of “classical” EAE or “complex EAE”

(C57Bl/6J×SJL/J)F_1_ mice were injected subcutaneously at one site in the flank with 200 µl of emulsion containing PLP139-151 (100 µg), or rhMOG (200 µg) in CFA containing 300 µg Mycobacterium tuberculosis H37Ra (“classical” EAE). Active “Complex EAE” was induced by injecting 200 µl of emulsion containing a mixture of different encephalitogenic peptides, hMOG34-56, hPLP139-151, hMOBP15-36, hMBP89-104, hOSP55-80 (75 µg of each peptide) in CFA as above, or with 6 µg MSCH in CFA containing 250 µg Mycobacterium tuberculosis H37Ra. Mice received 300 ng pertussis toxin in 500 µl PBS in the tail vein immediately and 48 h after immunization. Following the encephalitogenic challenge, mice were observed and scored as previously described [37], [38].

### Adoptive transfer of “classical” EAE or “complex EAE”

Cell transfer experiments were conducted as previously described [4], [36]. Briefly, irradiated (400 rads) naive syngeneic BSF1 mice were injected i.v. with pathogenic line T cells specific for a single encephalitogenic epiotpe (“classical” EAE), or with a mixture of five different line T-cells, each specific for a different encephalitogenic myelin epitoe (hMOG34-56, hPLP139-151, hMOBP15-36, hMBP89-104, hOSP55-74) (“complex EAE”). The line T-cells were activated with their specific peptide for three days prior to transfer, as previously described [4], and the number of cells injected is indicated in the legend to figures. Mice were observed and scored as previously described [37].

### Cytokine analysis

IL-2, IFN-g, IL-4, and IL-10, were measured by ELISA according to standard protocols from PharMingen (San Diego, CA), as described previously [39]. The capture antibodies were rat anti-mouse IL-4 (18191D; PharMingen), rat anti-mouse IL-2 (18161D; PharMingen), rat anti-mouse IL-10 (AMC0102; BioSource International, Camarillo, CA,) and rat anti-mouse IFN-γ (AMC4834; BioSource International). The biotinylated antibodies used were rat anti-mouse IL-4 (18042D), rat antimouse IL-2 (18172D), rat anti-mouse IL-10 (18152D) and rat anti-mouse IFN-γ (18112D; all from PharMingen). IL-17 was measured by ELISA using a DuoSet ELISA Development kit (DY421; R&D Systems, Inc., Minneapolis, MN). TGF-b was measured by ELISA according to the standard protocol from R&D Systems (Minneapolis, MN), using recombinant human TGF-ß sRII/Fc chimera as capture reagent (341-BR; R&D Systems) and biotinylated anti-human TGF-ß1 antibody (BAF240; R&D Systems). Recombinant human TGF-ß1 (240-B; R&D Systems) was used to construct the standard curve.

### Flow cytometric analysis

The flurochrome labeled monoclonal antibodies, PE conjugated anti mouse CD152 (murine CTLA-4), and APC conjugated anti mouse CD4, were purchased from BioLegend and used according to the manufacturer's protocols. Cells were analyzed on Cytomics FC 500 system (Beckman Coulter) and analyzed by Beckman Coulter software. Isotype controls were routinely used in all the experiments.

## Results

### The generation and immunofunctional properties of the MS-related multi-epitope protein, Y-MSPc

Figure 1A shows the scheme for the construction of the synthetic gene encoding Y-MSPc. Y-MSPc was designed to encompass in tandem only rationally selected epitopes of each of the major encephalitogenic target myelin proteins relevant to MS, MBP, PLP, MOG, MOBP, and OSP. The Y-MSPc includes epitopes (Fig. 1A) that have been selected for each of the myelin proteins according to following criteria: reports on preferential reactivity against the epiopes by MS T-cells, and/or epitopes with encephalitogenic potential in laboratory animals, and/or according to bioinformatical data predicting registers of preferred binding to the MS-associated HLA-DRB1*1501 (HLA-DR15) and/or HLA-DQB1*0602 (HLA-DQ06). The rationale for selecting the epiopte clusters (shown in Fig. 1A) from each of the primary target antigens MOG, MBP, PLP, MOBP, and OSP is detailed in Text S1 (S1.1.). As described in Text S1 (S1.1.), each of the 20 epitope clusters present in Y-MSPc may contain several non-overlapping and overlapping myelin T-cell epitopes for HLA-DRB1*1501 and/or HLA-DQB1*0602 molecules of HLA-DR15, the most prominent haplotype among the Caucasian MS patients. Using structural bioinformatics we predicted that about 100 overlapping and non-overlapping potential myelin epitopes for (C57Bl/6JxSJL/J)F1 (BSF1) (I-A^b^×I-A^s^) mice are encompassed by the Y-MSPc [Text S1 - S.1.2.], containing the human myelin epitope clusters. It should be noted that the human myelin epitope clusters are highly homologous to the counterpart mouse myelin epitope clusters, and many of them are potential T-cell epitopes also for the mouse MHC. Epitope clusters within the Y-MSPc encompassing at least one major encephalitogenic epitope are depicted by two asterisks (Figure 1A). The Y-MSPc-encoding DNA and derived amino acid are shown in Fig. S1.

To study the immunofunctional properties of Y-MSPc, we first assessed the immunofunctional integrity of the epitopes artificially assembled within Y-MSPc, using antigen-specific T cell lines that were raised to peptides representing different encephalitogenic myelin epitopes encompassed within Y-MSPc. The pathogenic line T-cells specific to encephalitogenic epitopes of MBP, PLP, MOG, MOBP, and OSP (Table S1) were tested for their proliferative response to Y-MSPc as compared to their priming epitope-containing peptide. Results shown in Fig. 1B indicate that the relevant epitopes within Y-MSPc can be appropriately processed and presented by APCs to their respective specific line T cells, as all the different MBP-, PLP-, MOG-, MOBP-, and OSP-specific encephalitogenic line T cells (Table S1) that were derived from BSF1 mice proliferated in response to Y-MSPc stimulation.

The stimulation of different epitope-specific line T cells by Y-MSPc indicates that antigenic processing and presentation of the encompassed epitopes to specific T-cells were not affected by their being joined together in tandem.

### Y-MSPc vs. peptide in suppression of EAE induced by a single encephalitogenic myelin antigen/epitope

**Figure 2:**
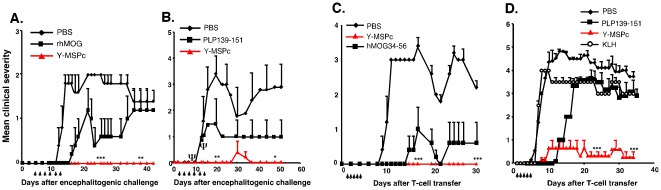
Systemic administration of Y-MSPc suppresses *“classical”* EAE induced by a single encephalitogenic antigen/peptide more effectively than peptide administration. (A, B), *Suppression of actively induced EAE by Y-MSPc vs. specific antigen/peptide*. BSF1 mice were immunized for induction of EAE by rhMOG (A) or PLP139-151 (B). On days indicated by arrows, the mice (n = 5 per group) received i.v. injection of soluble rhMOG (75 µg), PLP139-151 (75 µg), or Y-MSPc (75 µg) in 0.5 ml PBS, or PBS alone. **Ψ**, denotes mortality in the PLP139-151 treated group (Fig. 2B) due to anaphylactic shock on days 9 and 11 after immunization (one mouse and two mice, after the third and fourth injections, respectively), and therefore from day 11, the data are given for the two remaining mice only. (C, D), *Suppression of passive EAE by Y-MSPc vs. specific peptide*. EAE was passively transferred in naïve BSF1 mice with phMOG34-56-specific (C) or phPLP139-151-specific (D) line T cells (2×10^6^ cells). From days 1 to 5 (arrows), the mice (n = 6 per group) were injected i.v. with 75 µg of hMOG34-56, PLP-139-151, Y-MSPc, or KLH in 0.5 µl PBS, or with PBS alone. Shown are the clinical scores of one experiment out of two independent experiments that were performed of suppression of active (A, B) and passive (C, D) EAE. The two independent experiments yielded similar results, and the propensity of PLP peptide to induce high frequency of fatal anaphylactic shock was observed in the two experiments, as previously reported [52]. *, *p*<0.05; ** , *p*<0.005; ***, *p*<0.0005, compared to PBS control group; two tailed unpaired Student's t-test.

The efficacy of Y-MSPc in suppression of “classical” EAE (induced by a single encephalitogen/peptide) upon tolerogenic administration (soluble, i.v.) was assessed, and compared with tolerogenic administration of the relevant disease inducing antigen/peptide. As shown in Fig. 2, systemic administration of Y-MSPc before disease onset almost totally abrogated the development of EAE actively induced with rhMOG (Fig. 2A) or PLP139-151 (Fig. 2B), or EAE passively transferred with line T-cells specific for phMOG34-56 (Fig. 2C) or phPLP139-151 (Fig. 2D). Moreover, upon tolerogenic administration, the Y-MSPc was more effective than disease-inducing antigen/peptide in the suppression of active (Fig. 2A&B) or passive (Fig. 2C&D) EAE induced by MOG or PLP139-151. [The systemic administration of PLP139-151 consistently delayed, but did not suppress passive EAE induced in BSF1 mice by PLP139-151-specific T cells, for reasons not yet understood]. These data suggest that systemic administration of the disease relevant epitopes when contained within Y-MSPc is a more effective tolerogenic route than their administration as individual peptide (even though Y-MSPc was at lower molar ratio than each peptide ∼1∶20), and that Y-MSPc is more efficacious than the relevant peptide in the inhibition of already committed encephalitogenic T cells (in passive EAE, Figs. 2C&D). In addition, while Y-MSPc appeared devoid of adverse effects, the mice induced to develop active EAE with PLP139-151 and treated by PLP139-151 in PBS suffered from a hypersensitivity reaction, which resulted in death by anaphylactic shock (three mice died in the experiment presented in Fig. 2B; the results presented for this group are for the remaining 2 mice).

### Efficacy of Y-MSPc vs. peptide mixture as ‘multi-epitope targeting’ agents in suppression of “complex EAE”

**Figure 3:**
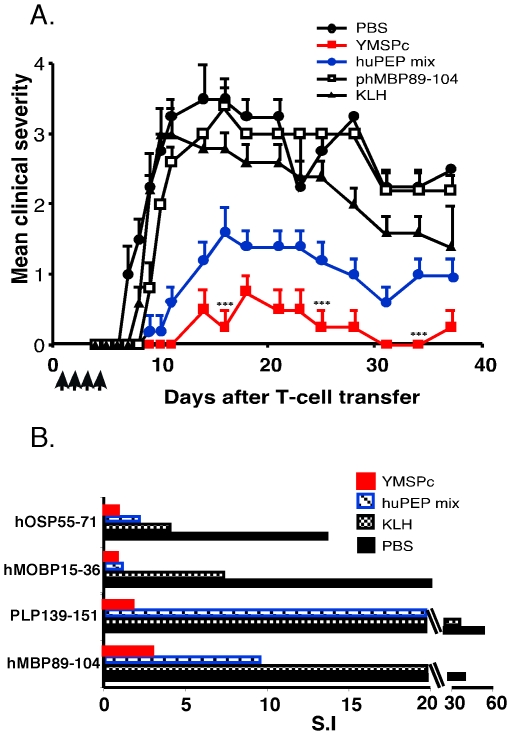
Y-MSPc is more efficacious than peptide cocktail (huPEP mix) in suppressing passively transferred “complex EAE” and in concomitantly downregulating multiple pathogenic anti-myelin T-cells. “Complex EAE” associated with multiple pathogenic anti-myelin T-cell autoreactivities was passively transferred to BSF1 mice with a pool of five activated encephalitogenic T-cell lines: Recipient mice were injected i.v. with 0.5 ml PBS containing phMBP89-104-specific line T-cells (0.5×10^6^), phPLP139-151-specific line T-cells (0.5×10^6^), phMOG34-56-specific line T-cells (0.7×10^6^), phMOBP15-36-specific line T-cells (0.8×10^6^), and phOSP55-74-specific line T-cells (0.5×10^6^). Each of the individual T-cell lines is highly encephalitogenic (Table S1). (A) *Efficacy of Y-MSPc vs. peptide cocktail (huPEP mix), as ‘multi-epitope targeting’ agents, in suppressing passively transferred “complex EAE”*. Recipients of the pool of encephalitogenic T-cell lines were injected i.v. daily, from days 1 to 4 after transfer, with phMBP89-104 (75 µg), a mixture of the 5 relevant peptides at equivalent amounts (huPEP mix, 150 µg total), Y-MSPc (100 µg), KLH (100 µg) as negative control, or with PBS, and followed for the development of clinical signs of EAE. (*B), Y-MSPc suppression of passively transferred “complex EAE” is associated with concomitant downregulation of each of the multiple encephalitogenic T-cells*. Recipients were injected on days 1, 2, 3, and 4 after T-cell transfer with Y-MSPc (100 µg), KLH (100 µg), huPEP mix (150 µg) in 0.5 ml PBS, or with PBS alone. On day 7, spleen cells were isolated and tested for their proliferative response to each of the relevant five peptides (10 µg/ml). The in-vivo and in-vitro suppression effects presented are from one experiment representative of two (A) or three (B) independent experiments. ***, *p*<0.0005 compared to PBS control group; two tailed unpaired Student's t-test.

We and others have shown that for EAE induced by several encephalitogenic myelin proteins/peptides, “complex EAE”, a treatment which targets autoreactivity against only one of these encephalitogens is not sufficiently effective in abrogating the development of the disease [20], [33] Since upon a definite diagnosis of MS, the disease is likely already associated with complex anti-myelin autoreactivity, due to “epitope spread” [26], [28], [31] , we assessed the efficacy of the multi-epitope-targeting approach, via Y-MSPc vs. mixture of relevant peptides, as multi-epitope targeting agents, in downregulating the multiple pathogenic anti-myelin autoreactivities, concomitantly. Encephalitogenic T-cell lines specific for phMBP89-104, phPLP139-151, phMOG34-56, phMOBP15-36, or phOSP55-74 were derived from BSF1 mice immunized with the relevant peptide (Table S1). “Complex EAE” associated with multiple pathogenic anti-myelin autoreactivies against five major encephalitogenic myelin proteins was induced by transfer of a pool of the five independent encephalitogenic T-cell lines into naïve BSF1 mice. Fig. 3A shows that tolerogenic administration of Y-MSPc was superior to single peptide (phMBP89-104) or even to a mixture of relevant peptides (huPEP mix) in suppressing the development of passive chronic “complex EAE”. While recipient mice that had been treated with PBS or with control protein keyhole limpet hemocyanin (KLH) developed severe chronic EAE (mean maximal clinical score of ∼3.5 or 3.0, respectively), the development of the disease in mice treated with Y-MSPc was strongly suppressed (mean maximal clinical score of 0.75) (Fig. 3A). Although treatment with soluble huPEP mix containing five peptides representing the epitopes of the five encephalitogenic T-cell lines also reduced disease severity, (mean maximal clinical score of 1.6), suppression by huPEP mix was significantly less effective than that by Y-MSPc. It should be also noted that the equivalent proportion of epitopic region in the Y-MSPc is considerably lower than that of each peptide in huPEP mix, i.e. molar ratio ∼7.5∶1 for individual peptide∶ Y-MSPc.

The insignificant effect of the systemic administration of only phMBP89-104 on the passively transferred “complex EAE” (Fig. 3A), further emphasize that targeting pathogenic autoimmune cells reactive against only a single epitope (peptide) is not sufficiently effective for treatment of a disease associated with multiple pathogenic anti-myelin autoimmunity.

Ex-vivo analysis following the different treatments (Fig. 3B) revealed that disease suppression was associated with downregulation of the transferred pathogenic line T-cells, and that the Y-MSPc was more effective than huPEP mix in downregulating the pathogenic T cells reactive against each of the myelin epitopes, concomitantly. Thus, splenocytes from BSF1 recipients of the five pathogenic T-cell lines that were treated with Y-MSPc showed over 90% reduction in the reactivity against the different relevant peptides, as compared to splenocytes from recipients treated with PBS, and over 75% reduction when compared to control treatment with KLH, which showed some non-specific, but yet significant, downregulation of anti-myelin autoreactivity (Fig. 3B). Interestingly, although treatment with huPEP mix also resulted in a concomitant downregulation of T cells reactive against the different myelin epitopes (25–35%, compared to PBS treatment, Fig. 3B), albeit less than that by Y-MSPc, the downmodulation of “complex EAE” by huPEP mix was only mild compared to that by Y-MSPc (Fig. 3A).

### Analysis and comparison of the anti-PLP peripheral regulatory mechanisms induced by treatment with Y-MSPc vs. single PLP peptide or huPEP mix

**Figure 4:**
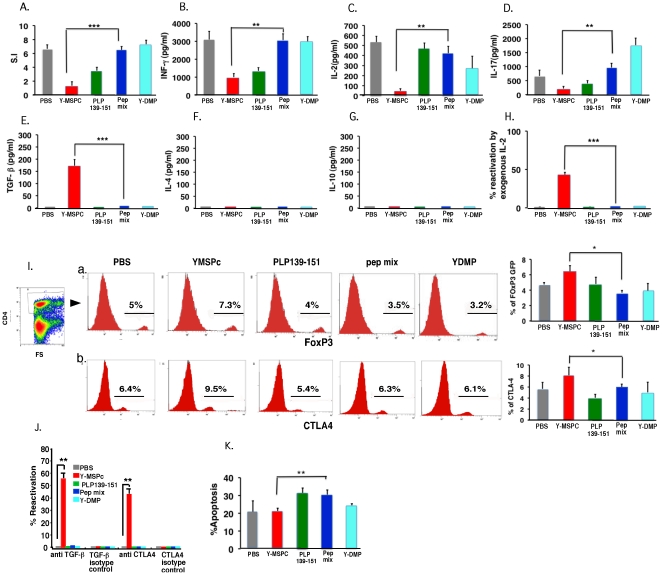
Analysis of the efficacy of anti-PLP peripheral regulatory mechanisms induced by treatment with Y-MSPc vs. single PLP peptide or peptide mixture (huPEP mix). (C57Bl.FoxP3GFPxSJL/J)F1 mice were immunized s.c. with PLP139-151 (100 µg) in CFA. On day 3, 5, and 7 post immunization, mice (n = 3/group) were injected i.v. with YMSPc (75 µg/mouse), PLP139-151 (100 µg/mouse), huPEP mix (total 140 µg/mouse), Y-DMP (75 µg/mouse), or with 0.5 µl PBS. The huPEP mix contained 1∶1 ratios of phPLP139-151, phPLP175-194, phMOG34-56, phMBP89-104, phMOBP15-36, phOSP55-80 and phOSP186-205 (20 µg each peptide), representing the major encephalitogenic epitopes of five human myelin proteins. A control treatment with non-relevant recombinant protein (Y-DMP) was also included to exclude the possibility that residual bacterial contaminants contributed to the efficacy of Y-MSPc. (Y-DMP is a diabetes mellitus-related recombinant artificial protein encompassing selected multiple epitopes of several antigens related to diabetes; The Y-DMP was constructed, expressed and purified similar to Y-MSPc). Three days later (10 days after immunization), draining LN cells from each treatment group were pooled and analyzed for: (**A**), *Ex vivo recall proliferative response to PLP139-151*. The LN cells from each treatment group were cultured for 72 h in microtiter wells in triplicates (0.5×10^6^/well) in the absence or presence of PLP139-151 (5 µg/ml ). [H^3^]Thymidine was added for the last 18 h. *(* ***B–G*** *), Secretion of pro-inflammatory (B–D) and anti-inflammatory (E–G) cytokines*. LN cells from each treatment group were cultured (5×10^6^/ml ) in triplicate cultures in the absence or presence of PLP139-151 (5 µg/ml ) for 48 h, and the supernatants were collected for measuring the secreted INF-γ, IL-2, IL-17, TGF-ß,, IL-4 and IL-10 cytokines by ELISA. For each treatment group, the net values are presented (after subtracting the values obtained in control cultures without PLP peptide). (**H**), *Ex-vivo recall proliferative response to PLP139-151 in the presence of exogenous IL-2*. LN cells from the different treatment groups were cultured in microtiter wells in triplicates without or with PLP139-151 (5 µg/ml) [as in (A)] and in the absence or the presence of exogenous rIL-2 (3.5 U/ml) for 72 h, with [H^3^]Thymidine added for the last 18 h. Calculating % reactivation: For each treatment group, the S.I. calculated for the recall proliferative response in the presence of exogenous IL-2 was divided by the S.I. calculated in the absence of exogenous IL-2. (**I**), *Flow cytometry of regulatory T-cells induced following systemic administration of YMSPc, PLP139-151, huPEP mix, Y-DMP, or PBS*. Draining LN cells from different treatment groups were co-stained with anti-CD4-APC and anti-CTLA-4-PE. The percentage of the FoxP3 (a) or CTLA-4 (b) expressing cells on gated CD4+ cells is shown. The FACS histograms are from one representative experiment, and the panels at the right end of Ia and Ib are the mean values +/−SD from three independent experiments. (**J**), *Ex-vivo recall proliferative response to PLP139-151 by primed LN cells in the presence of neutralizing anti- TGF-ß*, *or anti-CTLA-4 antibodies.* Draining LN cells from the different treatment groups were cultured without or with PLP139-151 (5 µg/ml) [as in (A)] and without or with added neutralizing antibodies anti- TGF-ß, or anti-CTLA-4 *(10 µg/ml)*, or respective isotype control antibodies. Calculating % reactivation: The S.I. calculated for the recall proliferative response in the presence of neutralizing antibodies or the respective isotype control antibodies was divided by the S.I. of the recall response in the absence of neutralizing or isotype control antibodies, respectively. (**K**), *Percent apoptotic cells in CD4+ T cell population of different treatment groups*. Apoptosis was determined by FACS analysis of Annexin V/7-AAD staining on gated CD4+ cells. The data presented in A, H, I (the panels a and b at the right end), J, and K, are the mean values from three independent experiments. Data presented in B–G are the mean +/−SD of triplicate cultures of pooled LN cells from one experiment representative of three independent experiments showing a similar pattern. The significance of the effect of Y-MSPc treatment compared to PBS control treatment was *p* = 0.0003 in A; p = 0,001 in B: p = 0.0001 in C: p = 0.02 in D: p = 0.0002 in E: p<0.00001 in H; p = 0.03 in Ia: p = 0.04 in Ib; and p = 0.05 in K. The significance of the effect of Y-MSPc treatment compared to huPEP mix treatment: *, *p*<0.05; **, *p*<0.005; ***, *p*<0.0005 (two tailed Student's t-test).

The efficacy of the regulatory mechanisms induced following treatment of PLP139-151-EAE by tolerogenic administration of the disease inducing peptide (PLP139-151) vs. peptides mixture (huPEP mix) or Y-MSPc, was elaborated using (C57Bl.FoxP3GFPxSJL/J)F1 mice. These mice are as susceptible to induction of EAE as the wild-type BSF1 mice (data not shown). PLP139-151/CFA immunized mice were treated by i.v. injections of soluble phPLP139-151, Y-MSPc, huPEP mix, Y-DMP, or PBS, on days 3, 5, and 7 post immunization. The huPEP mix (containing seven encephalitogenic peptides representing the major encephalitogenic epitopes of five human myelin proteins) and the non-relevant control recombinant protein, Y-DMP, are defined in legend to Fig. 4. Three days after last injection (day 10 post immunization), the effects of the various treatments on PLP139-151-reactive T-cells in the draining LN were analyzed ex-vivo. Fig. 4 shows that systemic administration of Y-MSPc was more potent than treatment with PLP139-151 or with huPEP mix in the induction of specific anti-PLP peripheral tolerogenic mechanisms, by most parameters analyzed. Thus, while Y-MSPc significantly reduced the recall proliferative response to PLP139-151 by about 80%, compared to PBS, treatment with pPLP139-151 reduced the response by about 50%, whereas the effect of the treatment by huPEP mix was only marginal (Fig. 4A). The downregulation of the PLP response following treatment with Y-MSPc was associated with strong reduction in the secretion of the proinflammatory cytokines, INFg (about 70%; Fig. 4B), IL-2 (about 90%; Fig. 4C), and IL-17 (about 70%; Fig. 4D) compared to PBS treatment. PLP139-151 treatment also reduced secretion of IFN-γ and IL-17 (about 55% and 40%, respectively, compared to PBS treatment), albeit consistently less than that following Y-MSPc treatment. In contrast, treatment with huPEP mix was by far less effective (in several experiments) in reducing the Th1/Th17 cytokines (Figs. 4B,C&D). As shown in Figs. 4F&G, neither Y-MSPc treatment nor PLP139-151 or huPEP mix treatment elevated IL-4 or IL-10 anti-inflammatory cytokines, at this time point of analysis (3 days after treatment; see analysis below on day 7 after treatment for comparison). This pattern of cytokine secretion was consistent in the three independent experiments that were carried out.

Interestingly, addition of exogenous IL-2 to the microcultures enhanced the recall proliferative response to PLP139-151 by the Y-MSPc-treated, but not by PLP139-15-, huPEP mix-, or PBS-treated primed LN cells (Fig. 4H), suggesting that only treatment by Y-MSPc induced a state of anergy in PLP139-151-reactive T-cells. In contrast, analysis of 7AAD/Annexin-V staining of CD4+ LN cells from Y-MSPc- vs. PLP139-151- or huPEP mix-treated mice, revealed that systemic administration of phPLP139-151 or huPEP mix, but not Y-MSPc, significantly elevated the apoptosis by about 10% over the PBS treatment (p<0.05) and over Y-MSPc treatment (p<0.005) Fig. 4K). However, as shown in Fig. 4E, only primed LN cells from Y-MSPc-, but not phPLP139-151- or huPEP mix-, treated mice secreted significant amount of TGF-ß, suggesting that downregulation of PLP139-151-reactive T-cells by Y-MSPc may involve induction of regulatory T-cells. Indeed, FACS analysis of PLP139-151-primed LN cells from mice treated with PBS, Y-MSPc, PLP, or huPEP mix, showed that 7.3% of the CD4+ LN cells from Y-MSPc-treated mice were FoxP3+ cells, compared to 4%, 3.5%, or 5% FoxP3+ cells of total CD4+ T-cells from phPLP139-151-, huPEP mix-, or PBS-treated LN cells, respectively (Fig. 4Ia histograms). Similarly, in the same experiment, 9.5% of the CD4+ T-cells of LN cells from Y-MSPc-treated mice were CTLA-4+ cells, compared to 5.4%, 6.3%, or 6.4% CTLA-4+ cells of total CD4+ T-cells from phPLP139-151-, huPEP mix-, or PBS-treated LN cells, respectively (Fig. 4Ib histograms). The increase in Foxp3+ and CTLA-4+ cells following treatment with Y-MSPc was consistent in all the three independent experiments that were carried out (Figs. 4Ia&b, right end panels). The results (Fig. 4J) showing that the neutralizing antibodies anti-TGF-ß or anti-CTLA-4 increased by about 55% or 45%, respectively, the recall proliferative response to PLP139-151 by primed LN cells only from Y-MSPc-treated, but not from PLP139-51-, huPEP mix-, Y-DMP, or PBS-treated mice (Fig. 4J), indicated the functionality of the Foxp3+/CTLA-4+ Treg cells, and further supported the possibility that suppression of EAE by treatment with Y-MSPc, but not with PLP139-151 peptide or with huPEP mixture of peptides, involves induction of CD4+ regulatory T cells.

The data presented in Fig. 4 strongly suggest that treatment with Y-MSPc is superior to treatment with PLP139-151 in the induction of anti-PLP peripheral regulatory mechanisms, explaining the higher efficacy of Y-MSPc in the suppression of actively or passively induced classical EAE (Fig. 2). Also, the more effective anti-PLP peripheral regulatory mechanisms induced by Y-MSPc compared to huPEPmix, is commensurate with the higher efficacy of Y-MSPc vs. huPEPmix in the suppression of passively induced “complex” EAE (Fig. 3).

### Downregulation of pathogenic autoimmunity against PLP last longer following treatment with Y-MSPc than with a single peptide or peptide mixture

**Figure 5:**
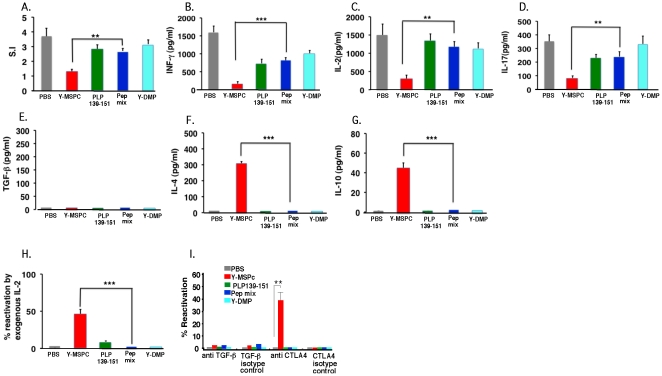
Systemic administration of Y-MSPc induces longer lasting peripheral regulatory mechanisms than PLP139-151 peptide or peptide mixture (huPEP mix). (C57Bl.FoxP3GFPxSJL/J)F1 mice were immunized with PLP139-151/CFA and injected i.v with YMSPc, PLP139-151, huPEP mix, Y-DMP, or with PBS, on day 3, 5, and 7 post immunization, as in Fig. 4. Seven days after last injection (14 days after immunization), splenocytes from each treatment group (n = 2/group) were pooled and analyzed as detailed for the LN cells in Fig. 4, for: (**A**), *Ex vivo recall proliferative response to PLP139-151 (5 µg/ml)*; (**B–G**), *Secretion of pro- and anti-inflammatory cytokines*; (**H, I**), *Ex-vivo recall proliferative response to PLP139-151 as in (A) but in the absence or presence of exogenous IL-2 (H), or neutralizing antibodies anti-TGF-ß or anti-CTLA-4 (I), or their respective isotype control antibodies*. The ex-vivo analysis of the responses of splenocytes was carried out as detailed in the legend to Fig. 4, respectively. The data presented in A, H, and I, are the mean +/−SD values from three independent experiments. Data presented in B–G are the mean +/−SD of triplicate cultures of pooled splenocytes from one experiment representative of three independent experiments showing a similar pattern. The significance of the effect of Y-MSPc treatment compared to PBS control treatment was *p* = 0.002 in A; p = 0,0001 in B; p = 0.002 in C: p = 0.0009 in D: p<0.0001 in F: p = 0001 in G&H. The significance of the effect of Y-MSPc treatment compared to huPEP mix treatment: *, *p*<0.05; ** , *p*<0.005; ***, *p*<0.0005 (two tailed Student's t-test).

The efficacy of anti-PLP peripheral regulatory mechanisms was assessed also 7 days after the last tolerogenic administration of the different agents. Splenocytes from mice that were immunized with PLP139-151/CFA and treated with Y-MSPc, phPLP139-151, huPEP mix, Y-DMP, or PBS (as in Section 3.4), were analyzed (on day 14 post immunization and 7 days after last treatment) for their ex-vivo recall proliferative responses against PLP139-151, and for their cytokine secretion pattern, as above. The results presented in Fig. 5 show that the downregulation of PLP-reactive T-cells following treatment with Y-MSPc remained quite potent (about 70% compared to PBS treatment) also 7 days after last systemic administration (Fig. 5A), while downregulation following treatment with phPLP139-151or huPEP mix was only about 23% and 27%, respectively, compared to PBS treatment) (Fig. 5A). Although the treatment with Y-MSPc, as well as with phPLP139-151 or huPEP mix reduced secretion of IFN-γ and IL-17 to a significant levels (Figs. 5C&D), the secretion of IFN-γ, IL-17 and IL-2 was more profoundly reduced following treatment with Y-MSPc (75–90%), compared to that following treatment with phPLP139-151 or huPEP mix (Figs. 5C&D). In addition, contrary to the results obtained with LN cells three days after last systemic administration (Fig. 4F&G), the secretion of IL-4 and IL-10 anti-inflammatory cytokines became significantly elevated, but only by primed PLP-reactive splenocytes from mice treated by Y-MSPc, but not by phPLP139-151 or huPEP mix (Figs. 5F&G). Moreover, only primed PLP-reactive T-cells from mice treated by Y-MSPc could be reactivated ex-vivo in the presence of exogenous IL-2 (Fig. 5H), indicating that PLP-reactive T-cells are still under anergy also 7 days after cessation of Y-MSPc treatment. As shown in Fig. 5I, the presence of neutralizing anti-CTLA-4, but not anti-TGF-ß, antibodies increased the proliferative response of splenocytes only from Y-MSPc-treated mice, suggesting that CTLA-4-associated regulation is still operative 7 days after last treatment with Y-MSPc. However, unlike 3 days after cessation of treatment (Fig. 4Ia&Ib), we could not observe a significant elevation of FoxP3+ or CTLA-4+ cells in the spleen of Y-MSPc-treated mice, compared to PLP-, huPEP mix-, treated mice (data not shown), or secretion of TGF-ß, by the primed splenocytes (Fig. 5E). Also contrary to 3 days after treatment, a significant increase in apoptosis could not be detected in splenocytes of any of the treatment groups by day 7 after cessation of treatment (data not shown).

Overall, the results of Figs. 4&5 altogether show that following systemic administration, Y-MSPc was clearly more effective than PLP139-151 or huPEP mix in inducing peripheral tolerogenic mechanisms that resulted in a more profound downregulation of pathogenic PLP139-151-reactive T-cells. These results also indicated that the immunomodulatory effect of Y-MSPc could not be attributed to residual bacterial components, as treatment with the control recombinant Y-DMP had no effect on downregulation of PLP139-151-reactive T-cells (Figs. 4A&5A). While downregulation of PLP139-151-reactive T-cells following treatment with PLP139-151 was more associated with induction of apoptosis, Y-MSPc treatment was associated with induction of anergy and FoxP3+/CTLA-4+ regulatory T-cells (Figs. 4&5), and with shifting the cytokine secretion profile of PLP-reactive T-cells from Th1/Th17 pro-inflammatory to Th2 anti-inflammatory cytokines (IL-4, IL-10) (Fig. 5). Moreover, the regulatory mechanisms induced following treatment with Y-MSPc were longer lasting than that induced by PLP139-151 or peptide mixture (Fig. 5).

### Reversal by Y-MSPc of ongoing EAE induced by pathogenic autoimmunity against a single encephalitogenic protein/epitope

**Figure 6:**
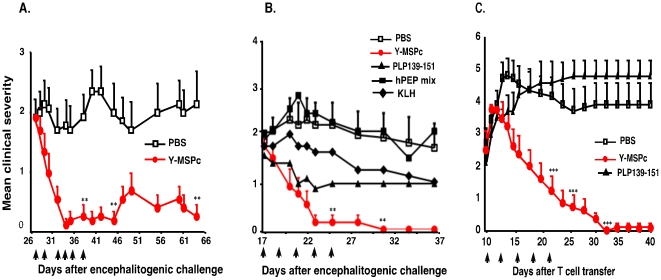
Treatment with Y-MSPc is more effective than peptide(s) in reversing ongoing “classical” EAE actively or passively induced by PLP139-151. BSF1 mice were immunized with phPLP139-151/CFA or were infused with syngeneic encephalitogenic PLP139-151-specific line T cells (2×10^6^ per mouse) for the development of actively or passively induced chronic EAE, respectively. (**A**), *The therapeutic effect of Y-MSPc on chronic EAE is long lasting*. On day 27 after immunization, mice with established chronic EAE (about two weeks after disease onset) were grouped (with equal mean clinical score of ∼2/group; n = 7 mice/group) and treated on days indicated by arrows by i.v. injections of Y-MSPc (75 µg in 0.5 ml PBS) or PBS alone. (**B**), *Reversal by Y-MSPc vs. phPLP139-151 or huPEP mix of actively induced ongoing EAE*. On day 17 after immunization, mice with ongoing EAE (about a week after disease onset) were grouped (with equal mean clinical score of ∼2/group; n = 7 mice/group) and treated on days indicated by arrows *by* i.v. injections of Y-MSPc (75 µg), phPLP139-151 (100 µg), huPEP mix (7 peptides as in Fig. 4; total 140 µg), KLH (75 µg), or PBS. (C), *Reversal by Y-MSPc vs. phPLP139-151 of passively induced ongoing EAE*. On day 10 after transfer, mice with ongoing EAE (4 days after onset) were treated by i.v. injections with 50 µg of PLP139-151, 75 µg of Y-MSPc, or PBS alone (n = 6 per group). Results are shown as mean clinical score +/−SE. **, *p*<0.005; ***, *p*<0.0005 compared to PBS control group; two tailed Student's t-test. P = 0.1 (n.s.) for the KLH treatment group, compared to PBS, for days 28 and 31.

While systemic administration of Y-MSPc strongly suppresses disease development (Figs. 2&3), potential approaches to therapy of MS are only worth considering if their efficacy extends to treatment of ongoing disease. We therefore assessed the effect of the treatment by Y-MSPc on the clinical course of ongoing chronic EAE induced by pathogenic autoimmunity against a single myelin epitope/antigen. BSF1 mice with established chronic EAE induced by active immunization with PLP139-151 were treated (10–12 days after disease onset) every 2–3 days with Y-MSPc or PBS. As shown in Fig. 6A, tolerogenic administration of Y-MSPc resulted in an immediate disease amelioration that progressed to almost a full recovery that lasted until the experiment was terminated (over three weeks after cessation of treatment). In contrast, 6 of the 7 mice treated with PBS showed a persistent chronic clinical EAE until the experiment was terminated (Fig. 6A).

The efficacy of Y-MSPc treatment was than compared with that of PLP139-151 peptide or huPEP mix in reversal of ongoing EAE, actively induced by PLP139-151. As shown in Fig. 6B, systemic administration of PLP139-151 peptide only arrested disease progression and moderately reduced the clinical manifestations. Rather intriguingly, the administration of huPEP mix had no effect on disease progression, while the administration of Y-MSPc resulted in an immediate disease amelioration and quick reduction in the clinical manifestations that progressed to a complete recovery.

A similar therapeutic efficacy of Y-MSPc emerged also following treatment of ongoing passive EAE transferred by committed pathogenic PLP139-151-specific T cells (Fig. 6C). In contrast, the systemic administration of PLP peptide (50 µg/injection) had no significant effect on the clinical course of the disease. However, as mentioned above, the effect of systemic administration of PLP139-151 on passively transferred PLP139-151-specific T cells into BSF1 mice is consistently remarkably low, for reasons not yet understood. Administration of higher amount of PLP139-151 peptide (100 µg/injection) resulted in high frequency of acute hypersensitivity reaction (data not shown).

### Treatment of chronic “complex EAE” by Y-MSPc vs. peptide cocktail, as “multi-epitope targeting” agents

**Figure 7:**
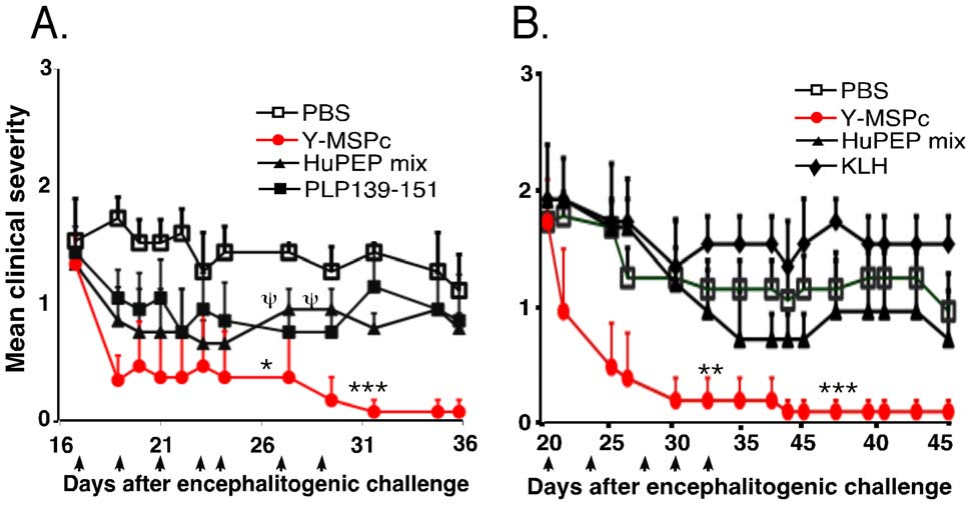
Efficacy of Y-MSPc vs. huPEP mix in reversal of ongoing “complex EAE” associated with multiple potentially pathogenic autoreactivities. (**A**), *Treatment of “complex EAE” induced by mixture of encephalitogenic peptides*. BSF1 mice were immunized with huPEP mix emulsified in CFA [a mix of five encephalitogenic peptides, phMOG34-56, phMBP89-104, phPLP139-151, phMOBP15-36 and phOSP55-80, (75 µg each)]. From day 17 after immunization, the mice (n = 5/group) were treated on days indicated by the arrows by i.v. injection with Y-MSPc (75 µg), PLP139-151 (100 µg), or huPEP mix (30 µg of each of the five peptides) in 0.5 ml PBS, or with PBS alone. **Ψ**, denotes mortality in the huPEP mix-treated group. Two mice died of anaphylactic shock on day 27 and day 29 after the sixth and seventh injection, respectively; data shown after day 29 are for the remaining 3 mice. (**B**), *Treatment of “complex EAE” induced by MSCH*. BSF1 mice were immunized with MSCH as described in Methods. From day 20 after immunization, mice with ongoing EAE (n = 5/group) were treated on days indicated by the arrows by i.v. injection with Y-MSPc (75 µg) in 0.5 ml PBS, huPEP mix (7 peptides as in Fig. 4; total 140 µg), KLH (75 µg) or with PBS alone. Results are shown as mean clinical scores +/−SE; *, *p*<0.05; **, *p*<0.005; ***, *p*<0.0005 compared to PBS control group; two tailed unpaired Student's t-test.

Upon definite diagnosis of MS, multiple pathogenic autoreactivities to myelin antigens may already be at play in patients. It is therefore essential to determine whether or not the therapeutic effect observed with ongoing EAE induced by autoreactivity to a single myelin epitope is effective also for ongoing “complex EAE” associated with multiple anti-myelin pathogenic autoimmunity. Chronic “complex EAE” was induced in BSF1 mice by immunization with a mixture of five (hMOG34-56, hMBP89-104, hPLP139-151, hMOBP15-36 and hOSP55-80) peptides (huPEP mix), each representing encephalitogenic epitope of five different human myelin proteins represented in the Y-MSPc. In this model, the expected pathogenic autoimmunity against the immunizing well-defined encephalitogenic epitopes (peptides), may be further complicated by emerging neo-autoreactivities against additional myelin epitopes, due to “epitope spread”. Mice with ongoing “complex EAE” were treated with Y-MSPc. PLP139-151, huPEP mix, or with PBS. As shown in Figure 7A, treatment with Y-MSPc resulted in a rapid reversal of clinical signs of EAE to almost complete recovery, compared to the consistent clinical signs of EAE in PBS-treated mice. In contrast, only moderate, albeit clear, beneficial effect was observed upon treatment with PLP139-151 alone or huPEP mix, however, two out of five mice treated with huPEP mix died of anaphylactic shock (Fig. 7A). The efficacy of Y-MSPc in treatment of “complex EAE” was also highly effective for ongoing “complex EAE” induced by immunization with MSCH, which contains all possible myelin and non-myelin CNS antigens. Fig. 7B shows that tolerogenic administration of Y-MSPc into mice with persistent clinical signs of EAE resulted in almost complete reversal of the clinical impairments, and the clinical amelioration perdured. In contrast, treatment with huPEP mix had only a marginal effect on disease progression (Fig. 7B).

**Figure 8:**
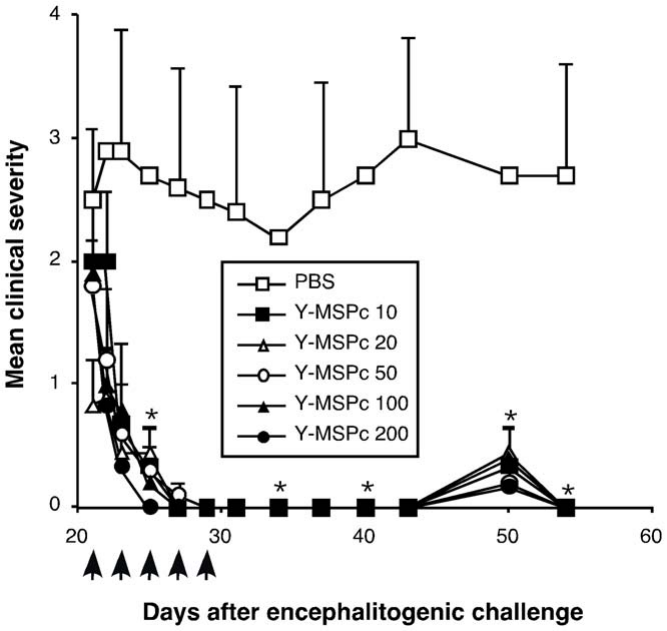
Effective reversal of ongoing PLP139-151-induced EAE by treatment with low doses of Y-MSPc. BSF1 mice were induced to develop “classical” EAE with PLP139-151. Starting on day 21 after immunization (6–8 days after disease onset), mice with a clinical score of at least 2 were grouped into groups with equal mean clinical score (5 mice/group) and treated with indicated doses of Y-MSPc in PBS (0.5 ml) or with PBS alone on days indicated by the arrows. Results shown are the mean clinical score +/−SE of one representative experiment out of two independent experiments showing a similar pattern; *, *p*<0.05 compared to PBS control group; two tailed Student's t-test.

Not less importantly, in the context of potential clinical utility of an antigen-based immune-specific therapeutic approach that is based on “multi-epitope targeting” agents, the Y-MSPc was highly effective even in very small quantities. Thus, as demonstrated in Fig. 8, tolerogenic administration of Y-MSPc resulted in almost complete reversal of ongoing PLP139-151/CFA-induced EAE even at doses as low as 10 µg (Fig. 8).

## Discussion

It is now widely accepted that the pathogenic autoimmunity in MS, as well as in other organ-specific autoimmune diseases, can be directed against several target antigens. In view of the multiplicity of potential target antigens in MS, the primary target antigen may be different in different patients. Moreover, in the same patient, the pathogenic autoimmunity may also shift or expand to other CNS target antigens with disease progression due to “epitope spread” [28], [31], [40]. Thus, it is likely that upon definite diagnosis, a complex pathogenic autoimmune process is already at play. In this study, data from experiments in different mouse models of MS show that antigen-based treatment with a single antigen/epitope(peptide) is unlikely to be a sufficiently effective therapy for organ-specific autoimmune diseases associated with complex pathogenic autoimmunity. Furthermore, and of major significance for immune-specific therapy, our data also strongly suggest that antigen-based therapy of organ-specific autoimmune diseases, such as MS, is likely to be effective only if the multiple pathogenic autoimmune T cells reactive against major organ-specific target antigens/epitopes would be concomitantly targeted. Thus, in “complex EAE”, a murine model that better resembles the complex pathogenic anti-myelin autoimmunity in MS, immune-specific treatment that targets pathogenic T cells reactive against only a single MOG or PLP epitope (peptide) is significantly less effective than treatment with a “multi-epitope targeting” agent that can concomitantly neutralize multiple pathogenic autoimmune T cells reactive against multiple myelin antigens/epitopes. Such a “multi-epitope-targeted” approach to immune-specific therapy for MS-like disease was investigated using the artificial multi-epitope protein, Y-MSPc, or a cocktail of human myelin peptides (huPEP mix), as “multi-epitope-targeting” agents. As presented here, treatment with Y-MSPc was consistently more effective than treatment with relevant peptide cocktail, both in suppressing the development of “complex EAE” and in ameliorating ongoing disease, via the induction of more efficacious and longer lasting peripheral regulatory mechanisms; and, of most significance for its potential clinical utility, the Y-MSPc was also more effective in the reversal of ongoing “complex EAE” associated with multiple pathogenic anti-myelin autoimmunity. These findings strongly suggest that concomitant targeting of multiple pathogenic T-cells is more effective when the multiple epitopes are encompassed within a specifically engineered globular protein rather than presented as a mixture of relevant synthetic peptides.

Notably, Y-MSPc was also consistently more effective in treatment of ongoing ”classical” EAE induced by a single encepalitogenic antigen/peptide (PLP139-151 or hMOG/hMOG34-56) than treatment with the disease-inducing peptide itself. The higher efficacy of Y-MSPc could be attributed, at least in part, to its inherent potential advantage in neutralizing also pathogenic neo-autoreactivities that could emerge from “epitope spread”. However, the systemic administration of Y-MSPc prior to disease onset (and before “epitope spread” emerged), suppressed “classical” EAE that was actively or passively induced by a single antigen/peptide (hMOG/hMOG34-56 or PLP139-151) more effectively than systemic administration of the relevant disease-inducing peptide. The superior efficacy of Y-MSPc in suppression of active “classical” EAE (Figs. 2A&B) was found to be related to its greater capacity than peptide(s) to induce peripheral tolerogenic mechanisms [anergy (Fig. 4&5), FoxP3+ CTLA-4+ regulatory T-cells (Fig. 4&5), and shifting of Th1/Th17 T-cells into anti-inflammatory Th2 cells secreting IL-4 and IL-10 cytokines; (Fig. 5)] resulting in more effective downregulation of PLP139-151-reactive T-cells in mice inoculated by PLP139-151/CFA for the development of EAE. In addition, the regulatory mechanisms induced following treatment with Y-MSPc, but not following treatment with a single peptide or peptide mixture, were still effective in downregulation of PLP139-151-reactive T-cells also 7 days after cessation of treatment. However, the peripheral regulatory mechanisms that were detected 3 or 7 days after cessation of treatment could not be detected any more 14 days after last treatment in the PLP139-151/CFA-immunized mice that were treated with Y-MSPc. Yet, compared to PBS-treated mice, the pathogenic T-cell reactivity against PLP in the spleen of mice treated with Y-MSPc remained quite low, almost at baseline levels, also after 14 days following cessation of treatment, as determined by analysis of their ex-vivo recall proliferative responses to PLP139-151 (data not shown). These data altogether, suggest that the peripheral regulatory mechanisms that develop during treatment with Y-MSPc are rather transient after cessation of treatment. However, the high efficacy of the Y-MSPc-induced regulatory mechanisms, albeit relatively “short-term” (>7 days and <14 days after cessation of treatment), was apparently sufficient to translate into longer lasting protection against the pathogenic PLP-reactive T-cells, as evident from the long lasting suppression and reversal of clinical EAE, weeks after cessation of treatment (Figs. 2&6).

The basis for the higher immunomodulatory effect of Y-MSPc compared to single peptide or peptide cocktail is, as yet, unclear. The likely higher degradation and clearance rate of peptide(s) compared to globular proteins could not be the sole explanation, since the treatment with a soluble peptide(s) versus Y-MSPc induced different regulatory mechanisms (Figs. 4&5). More efficient in-vivo uptake of Y-MSPc and different pathways of MHC-class II presentation, which favor presentation of particulate proteins over exogenous peptides [41], [42], is another plausible explanation for its higher immunomodulatory efficacy. The processing and presentation of antigens by immature dendritic cells or other non-activated APCs, such as B-cells or macrophages, can result in downregulation rather than upregulation of the antigen-specific immune response [43], [44]. Nonetheless, our results strongly suggest that upon systemic administration, peptidic myelin epitope(s) are less effective inducers of peripheral regulatory mechanisms than the same epitope(s) when incorporated within a globular multi-epitope protein, such as Y-MSPc. Why the multi-epitope protein is more effective than peptide in the induction of peripheral tolerogenic mechanisms and appears to operate via different tolerogenic mechanisms is quite an intriguing question and of high significance for a better understanding of peripheral antigen-specific tolerance as well as for antigen-based immunotherapy. Unfortunately, however, none of the published studies could provide experimentally based explanations, and the answer to this question is now under investigation.

Another multi-epitope targeting approach with splenocytes coupled with a peptide cocktail of four distinct encephalitogenic epitopes was recently reported to be effective in preventative tolerance as well as in the treatment of relapsing EAE [45]. In this study, the PLP139-151, PLP178-191, MBP84-104, and MOG92-106 chemically coupled to splenocytes were used as a “multi-epitope targeting” agent. The efficacy of this multi-epitope peptidic approach has not been compared with that of the specifically designed multi-epitope protein, Y-MSPc.

It has been known for several decades that administration of soluble MBP or the immunodominant peptides of MBP can prevent and treat MBP-induced EAE [19], [46] [47]. Similar observations have been made for prevention and treatment of PLP- and MOG-induced EAE [9], [20], [48]. The development of this immunospecific antigen/peptide-based treatment as an approach to therapy of MS or of other human autoimmune diseases was withheld for many years mainly because of the daunting inherent potential risk that administration of native antigen/peptide would also provoke pathogenic T cells and lead to disease exacerbation rather than remission. The injection of Y-MSPc in CFA could indeed induce EAE, albeit only with mild clinical manifestations (data not shown). However, Y-MSPc is highly tolerogenic when administered systemically as a soluble protein. In this context, it should be noted that following i.v. treatment of chronic EAE with soluble Y-MSPc, an immediate disease amelioration could be observed, while disease exacerbations were not observed in any of our experiments of suppression and treatment of chronic EAE by Y-MSPc (over dozen experiments). Moreover, and more significantly, in clinical trials with a soluble MBP, or with peptide (MBP8296) representing the major immunodominant epitope of MBP, infusions (i.v.) of MBP8296 or subcutaneous injection of MBP or transdermal application of myelin peptides did not cause disease exacerbations or any significant adverse effects in the treated patients [21], [22], [23], [49]. Nevertheless, the results of the clinical trials were disappointing [21], [22], [23]. Other clinical trials in MS with MBP altered peptide ligand (MBP-APL) also yielded disappointing results [24], [25]. All of these clinical trials, however, were designed to target pathogenic T-cells reactive against only a single target antigen/epitope without taking into consideration the complexity and dynamics of the pathogenic autoimmunity associated with MS. In fact, the failure of these clinical trials corroborates our concept of concomitantly targeting potentially pathogenic T cells reactive against multiple myelin target antigens/epitopes as an approach for the treatment of MS.

Of no less significance in our study is the general lack of hypersensitivity reaction upon treatment with Y-MSPc, while several mice died of anaphylactic shock following treatment with an individual peptide or peptide mixture encompassing encephalitogenic epitope(s), as was observed following treatment with PLP139-151 (Fig. 2B) or huPEP mix (Fig. 7A). The relatively frequent occurrence of anaphylactic reactions following treatment of EAE or type 1 diabetes with self peptides has been previously reported [50], [51], [52], and has been suggested to be related to the lack of thymic expression of the epitope encompassed within the relevant peptide [50]. These observations are likely to be of high relevance to the human disease, as shown in a trial for treatment of MS patients with an altered peptide ligand of MBP, which was terminated because of systemic hypersensitivity reactions to the peptide [24], [25]. In contrast, only negligible hypersensitivity reactions were observed in MS patients treated with the glatiramer acetate (Copaxone) [53]. Obviously, it is of major advantage for its clinical utility that the Y-MSPc can reach effective reversal of ongoing EAE by therapeutic doses that do not induce anaphylaxis. Why hypersensitivity reactions to peptides are far more frequent than to a globular polypeptide-such as glatiramer acetate or Y-MSPc is not yet known. However, since the hypersensitivity to peptide is IgE-dependent [54], the differences in hypersensitivity responses could be related to higher production of epitope-specific IgE following peptide treatment, and/or to a higher availability of the peptidic epitope for cross linking the specific IgE bound to FcεRI on mast cells.

Obviously, since not all potential target antigens have already been identified, and due to the unpredictable pattern of “epitope spread”, generating a Y-MSPc-like multi-epitope protein that can target pathogenic T-cells reactive against all potentially pathogenic antigens/epitopes in MS is not possible. However, assuming that after decades of intensive investigation by numerous laboratories worldwide, most of the major encephalitogenic target antigens in MS have been defined, it is likely that ‘cumulative’ specific regulatory mechanisms induced by the different major MS-relevant epitopes encompassed by the Y-MSPc-like multi-epitope protein may downregulate, via ‘cumulative bystander suppression’, also autoimmune T-cells reactive against other potential target (minor) antigens not represented within the “multi-epitope targeting” protein. In this context, it is of significance that Y-MSPc fully reversed EAE induced by MSCH, which contains all possible target antigens/epitopes of the CNS. In addition, the Y-MSPc could also reverse pre-established chronic EAE induced by single or multiple encephalitogenic peptides (Figs. 6&7), in which the “epitope spread”-related expansion of neo-autoreactivities is likely to already be at play.

Overall, our studies using models of “complex EAE” show that a concomitant “multi-epitope-targeting” approach is required for effective antigen-based immune-specific therapy of organ-specific autoimmune diseases associated with complex and dynamic pathogenic autoimmunity, such as MS. Our studies also favor the use of a specifically designed MS-relevant multi-epitope protein over cocktail of myelin peptides, as “mutli-epitope targeting” agents, for effective treatment of chronic MS-like disease. Such an artificial multi-epitope protein can be adapted to other organ-specific autoimmune diseases.

## Supporting Information

Text S1 **Selection of the myelin epitopes encompassed by the multi-epitope protein and construction of the synthetic gene encoding the multi epitope protein.**
(DOC)

Figure S1 **DNA sequence and expression of the Y-MSPc-encoding synthetic gene.** (A) The DNA and derived amino acid sequences of Y-MSPc were confirmed and shown to be in an open reading frame with the ATG of pRSET expression vector by DNA sequence analysis using pRSET-specific primers. (B) Bacterial expression of the Y-MSPc-encoding gene and isolation of Y-MSPc. Coomassie blue-stained SDS-PAGE analysis: Lane 1, molecular weight standards; lane 2, bacterial extract before IPTG induction; lane 3, after IPTG induction; lane 4, recombinant Y-MSPc isolated by metal-chelate affinity chromatography on Ni-NTA agarose (2 µg); lane 5, Ni-NTA-isolated Y-MSPc after high flow gel filtration on Superdex 75 (60 kd fraction; 3 µg). [The smaller and fainter bands (∼50 and 40 kd ) in lane 4 were shown to result of pre-mature termination of translation of the 60 kd Y-MSPc]
(TIF)

Table S1 **The encephalitpogenic potential of (C57Bl/6JxSJL/)F1-derived T-cell lines specific for different encephalitogenic myelin epitopes.** (C57Bl/6JxSJL/)F1 mice were injected s.c. with 100 µg of either peptide emulsified in CFA. T cell lines specific for the immunizing peptide were selected in-vitro from the draining LN cells that obtained 10 days after immunization, as previously described [7], [42]. Line T-cells at indicated numbers were injected i.v. into slightly irradiated (400 rads) syngeneic naïve recipients. Recipients were followed for development of clinical signs of EAE and scored as described [43].
(DOC)

Table S2 **List of myelin peptides used in this study.**
(DOC)
