# Supplementary material for: ‘Multi-Epitope-Targeted’ Immune-Specific Therapy for a Multiple Sclerosis-Like Disease via Engineered Multi-Epitope Protein Is Superior to Peptides
Source: PLoS One. 2011 Nov 29;6(11):e27860. doi: 10.1371/journal.pone.0027860 (PMC3226621; doi:10.1371/journal.pone.0027860)
Supplement: Text S1 — Selection of the myelin epitopes encompassed by the multi-epitope protein and construction of the synthetic gene encoding the multi epitope protein. (DOC) [file pone.0027860.s001.doc]

**Text S1 (Methods)**

**S1.1. Selection of the MOG, MBP, PLP, MOBP, and OSP epitopes included in the Y-MSPc**

The epitopes (epitope clusters) of each of the five known major encephalitogenic target myelin proteins in MS were selected according to the following criteria: reports of preferential reactivity by MS T-cells, and/or encephalitogenic potential in laboratory animals, and/or according to bioinformatical data predicting registers (described below) with preferred binding to the MS-associated HLA-DRB1*1501 (HLA-DR15) and/or HLA-DQB1*0602 (HLA-DQ06). The epitope selection was as follows:

**MOG epitope clusters:** We selected the epitope clusters for MOG to reside within amino acids 3-27, 34-56, 67-114, and 205-215, based on the following rationale: The MOG3-27 region contains epitopes preferentially recognized by MS T-cells[1,2]; a major T-cell epitope for rhesus monkeys with MOG-induced EAE[3]; and, epitopes with preferred binding mode to HLA-DR15 (residues 12-20) and HLA-DQ06 (residues 12-20 and 19-27) as predicted by structural bioinformatics analysis (detailed below). The MOG 34-56 region contains epitopes preferentially recognized by MS T-cells [1,2,4]; a major encephalitogenic T-cell epitope for rhesus monkeys with MOG-induced EAE [3,5]; the encephalitogenic epitopes for H-2b and H-2u mice [6,7,8] Lewis rats [9], and HLA-DR2 transgenic mice [10]; and, predicted epitopes with preferred binding mode to HLA-DR15 (residues 37-45 and 42-50) and HLA-DQ06 (residues 45-53). The MOG 67-114 contains epitopes preferentially recognized by MS T-cells [1,2,4]; a major T-cell epitope for rhesus monkeys with MOG-induced EAE [3]; the encephalitogenic epitope for SJL/J mice [11] and for HLA-DR4 transgenic mice [12]; and, predicted epitopes with preferred binding mode to HLA-DR15 (residues 69-77 and 82-90). MOG 205-215 region contains a predicted epitope with preferred strong binding mode to HLA-DR15 (residues 204-216) and a T-cell epitope for H-2u mice [8].

**PLP epitope clusters:** We selected the epitope clusters for PLP to reside within amino acids 38-52, 103-150, 177-203, 218-240 and 264-276: The PLP38-52 contains epitopes preferentially recognized by DR2 and D4 MS T-cells [13], one of the H-2d strains, BALB/cPt, is also moderately susceptible to EAE induced by PLP 40-59 [14]; The PLP103-150 contains epitopes that are more frequently recognized in MS [15,16] and shown to be associated with HLA-DRB1*1101 [17] as well as with DR2 [15,16], and two encephalitogenic epitopes that were delineated to amino acid residues 105-115 and 106-116 in SWR (H-2q) mice [18]. The PLP103-150 also overlaps with PLP91-120 that induced clinical EAE in DR3 Tg mice [19]; PLP177-203, contains epitopes associated to Dw2 alleles [17], encephalitogenic epitope for SJL/J (H-2s) mouse strain [18], and is also an encephalitogenic epitope for the two H-2d strains tested, BALB/cPt and BALB/cJ, as well as for two H-2k strains, AKR/J and C3HeB/FcJ [14]; PLP218-240, contains epitopes at the C-terminus (amino acids 220-276; include a cryptic epitope) have been reported as recognized in MS [20,21], albeit with no strong evidence of immunodominance in MS and the C3H/He (H-2k) mice have been reported to respond to amino acid residues 215-232 [22]. PLP215-232 induce also disease in the H-2k strain, CBA/J [14]; In DR2, DR3, DR4, DQ6 and DQ8 transgenic mice, the majority of the dominant cell epitopes were clustered mainly to three regions: 31-70, 91-120, and 178-228; All of epitope clusters mentioned above contained predicted epitopes with preferred binding affinity to HLA-DR15, including epitopes within PLP264-276.

**MBP epitope clusters:** We selected epitope clusters for MBP to reside within amino acids 12-42, 84-111 and MBP141-168: The MBP12-42 includes encephalitogenic epitope that induced severe EAE in HLA-DR1 Tg mice [23]. Marmosets immunized with human MBP reacted to phMBP11-30, and an epitope within MBP12-42 is encephalitogenic for SJL/J mice [24]; MBP84-111, includes an encephalitogenic epitope for H-2s mice and for Lewis rats [25,26]. An association of the immunodominant central epitopic region of MBP, within amino acids 80-105, to the DR2 haplotype, the major disease associated allele for MS, has been reported in several studies [27,28,29,30]. Also, T-cell response to MBP81-100 in HLA-DR3/DQ8 Tg mice with severe EAE was reported [31], and EAE with MBP84-102 was demonstrated in Tg mice which also expressed a human TCR associated with recognition of the MBP epitope [32]; MBP141-168, includes an encephalitogenic epitope for rhesus monkey [33], and marmosets immunized with human MBP reacted to epitope resides within MBP141-168.

**OSP epitope clusters:** We selected epitope clusters for OSP to reside within amino acids 21-34, 43-74, 99-110, 130-146 and OSP188-207: OSP21-34, is included in the minor encephalitogenic epitope in H-2^b^ mouse strain and is associated with a predominant B-cell response in this strain. OSP43-74, contains an encephalitogenic epitope (OSP55-71) for H-2^s^ mouse strain. OSP99-110, OSP130-146 and OSP188-207, contains epitopes detected in MS patients as well as normal controls, albeit a significantly higher percentage of patients with relapsing remitting [33]. OSP188-207 contains major encephalitogenic epitope for H-2b mouse strain and minor encephalitogenic epitope for H-2^s^ mouse strain.

**MOBP epitope clusters**: We selected epitope clusters for MOBP to reside within amino acids 15-33, 55-90 and MOBP156-172: The MOBP15-33 encompasses the immunodominant major encephalitogenic epitope for SJL/J mice and predicted epitope for multiple sclerosis-associated HLA-DRB1*1501 [34]. HLA-DQB1*0602-Tg mice and HLA-(DRB1*1501xDQB1*0602)F1 double Tg mice are susceptible to induction of EAE by MOBP, through pathogenic T-cells reactive against MOBP15-36 [35]; MOBP55-90, proliferative response to MOBP peptides by PBL from MS patients and controls indicated that MOBP65-68 was the most frequently recognized region by MS patients [36]. MOBP55-90 also contains a cryptic encephalitogenic epitope for H-2b mice [36]. MOBP55-77 harbor encephalitogenic epitopes for HLA-DQB1*0602-Tg mice and HLA-(DRB1*1501xDQB1*0602)F1 double Tg mice; and, predicted epitopes with preferred binding mode to HLA-DR15 (residue MOBP156-172).

Each of the epitope clusters composing the Y-MSPc may contain 1-4 non-overlapping T-cell epitopes for HLA-DRB1*1501 and/or HLA-DQB1*0602 molecules of HLA-DR15, the most prominent HLA haplotype among Caucasian MS patients. The same number of non-overlapping mouse I-A^b^ and/or I-A^s^ T-cell epitopes may be present in each of the epitope clusters of Y-MSPc. Notably, however, prediction of the number of potential DRB1*1501 and/or DQB1*0602-associated epitopes by structural bioinformatics methods (detailed below) shows that each of the epitope clusters may contain 1-14 overlapping DR-15- and/or DQ6-related epitopes. Thus, the Y-MSPc clusters were predicted to contain 103 potential overlapping and non-overlapping epitopes with a preferred binding affinity to DRB1*1501 and/or DQB1*0602: MOG epitope clusters were predicted to encompass 12 potential epitopes (4 DQ6- and 8 DR-related epitopes, some of which are overlapping DR/DQ epitopes); MBP epitope clusters were predicted to encompass 19 potential epitopes (11 DQ6- and 8 DR-related epitopes. some of which are overlapping DR/DQ epitopes); OSP epitope clusters were predicted to encompass 27 potential epitopes (8 DQ6- and 19 DR-related epitopes, some of which are overlapping DR/DQ epitopes); MOBP epitope clusters were predicted to encompass 9 potential epitopes (6 DQ6- and 3 DR-related epitopes, some of which are overlapping DR/DQ epitopes); and, the PLP epitope clusters were predicted to encompass 36 potential epitopes (12 DQ6- and 19 DR-related epitopes, some of which are overlapping DR/DQ epitopes); (data not shown). The number of the overlapping and non-overlapping epitopes within the Y-MSPc that were predicted by the structural bioinformatics to be with a preferred binding affinity to I-A^b^ and/or I-A^s^ molecules, is also ~100 epitopes. Thus, the Y-MSPc may contain ~100 overlapping and non-overlapping epitopes for BSF1 [(I-A^b^ x I-A^s^)F1] mice or for HLA-DR15^+^(DRB1*1501; DQB1*0602) MS patients, which comprise ~50-60 non-overlapping epitopes for I-A^b^&I-A^s^ or for DR15&DQ6 molecules.

**S1.2. Prediction of HLA-DR2-binding registers:**

Epitopes of MBP, PLP, MOG, MOBP, and OSP, with predicted preferred binding affinity to HLA DR15(DRA*0101/DRB1*1501) and/or to HLA DQ6(DQA1*0102/DQB1*0602) molecules of the HLA-DR15 haplotype (DR2 haplotype by old serological typing nomenclature) were selected for the construction of Y-MSPc. Many of these epitopes were detected by PBLs of MS patients or were encephalitogenic for laboratory animals. Predictions were carried out by Dr. M. Eisenstein, Department of Chemical Services, the Weizmann Institute of Science, as follows: Available structures of HLA-DR/DQ molecules and of MHC-II molecules were used to predict the preferred binding mode of nine-residue epitopes throughout each of the five encephalitogenic proteins, MBP, PLP, MOG, MOBP, and OSP. The crystal structure of the DR molecule (DRA*0101/DRB1*1501), deposited in the Protein Data Bank (PDB code 1bx2), and the crystal structure of the DQ molecule (PDB code IUVQ) were used to characterize the binding preferences of these MHC molecule. These were represented through a 9x20 matrix in which every entry is a number quantifying the preference of a given amino acid (20 possibilities) to be placed in a given position along the bound peptide (9 possibilities). A 9x20 binding preference matrix was also constructed for this molecule. The binding matrices were used in a computer program in which a 9 amino-acid moving window is applied to the sequence of a given protein and a binding score is calculated for each 9 amino-acid sequence. This score is estimated as the sum of the binding preferences from the appropriate 9x20 matrix. As one of the MHC structures (DQA1*0102/DQB1*0602) is modeled, it is less reliable than an experimental structure. Therefore, in order not to miss possible epitopes, the binding preference matrices must not be too strict. Sequences with binding scores above average were modeled in analogy to MHC class II-bound peptides in known structures (DR, I-Ak), and energy-minimized. Of these, sequences that were seen to fit into the MHC binding site were taken as representing possible HLA-binding epitopes. We have used this system successfully to predict I-Ab and I-As binding sequences of MOG [37], MOBP [38], and OSP [39], that were proven to be I-Ab or I-As -restricted T-cell epitopes. Our method is similar, albeit not as strict as the Artificial Neural Network [40].

**S1.3. Generation of Y-MSPc**

We first constructed the synthetic human multi-epitope minigenes, shMOG/MS, shMBP/MS, shOSP/MS, shMOBP/MS, and shPLP/MS (depicted in Fig. 1A) that were designed to encompass the rationally selected epitope clusters of human myelin proteins MOG, MBP, OSP, MOBP, and PLP, respectively, and than sequentially ligated the minigenes to generate the Y-MSPc synthetic gene encoding Y-MSPc protein (Fig. 1A), as detailed below:

**Oligonucleotides/primers related to generation of synthetic human myelin antigen minigenes.** For each of the minigenes (shMOG/MS, shMBP/MS, shOSP/MS, shMOBP/MS, and shPLP/MS), 60-70 nucleotide-long oligonucleotides, which represent codons of the amino acid residues of the selected epitopes aligned sequentially, and which are complementary at their 5' and/or 3' ends to their neighboring oligonucleotides by an overlap of 18 nucleotides, were synthesized by the Weizmann Institute Synthesis Unit. Relevant oligonucleotides include specific restriction endonuclease sites to enable cloning or in frame ligation to neighboring synthetic human autoantigen genes.

Synthetic oligonucleotides used for generation and amplification of **shMOG/MS** DNA: shMOG1a:**GAATTC**GCTAGCGGCATGGAGGTGGGGTGGTAT;shMOG1:GGCATGGAGGTGGGGTGGTATCGCCCACCATTCTCTAGGGTGGTCATCTCTACCGTAATGGCAAGGAC;shMOG2:(3'reverse)GAGAGTCACCTTTCCCTCACCAATAGCATCTTTCAGCAGCTCTGTACGGCCGTCCTTGCCATTACGGTA;shMOG3:GAGGGAAAGGTGACTCTCAGGATTCGGAATGTACGCTTCTCTGATGAAGGAGGTTTCACCAGCTTCTTC;shMOG4:(3'reverse)GAAAGATCTTTTCAATTCCATTGCTGCCTCCTCTTGGTAAGAATGGTCACGGAAGAAGCTGGTGAAACC;shMOG5:GAATTGAAAAGATCTTTCAGAGTGATAGGACCAAGACACCCAATCCGTGCTCTGGTCGGGGATGAAGTG;shMOG6:(3'reverse)CAGCTCTTCAAGGAATTGCCCTGCAAGTCTCGATATGCGAGATGGCAATTCCACTTCATCCCCGACCAG;shMOG6a:(3'reverse)**AAGCTT**TCAGGATCCACGCAGCTCTTCAAGGAATTGCCC. The NheI site in shMOG1a, the BglII in shMOG4 and shMOG5, and the BamHI site in shMOG6a are underlined. The EcoRI site in shMOG1a and the HindIII site in shMOG6a are bolded.

Synthetic oligonucleotides for generation and amplification of **shMBP/MS** DNA: shMBP1a:**GAATTC**GCTAGCGGATCCAACCCAGTAGTCCAC;shMBP1:GGATCCAACCCAGTAGTCCACTTCTTCAAGAACATTGTGACGCCACGCACACCACCACCGTCGCAGGGA;shMBP2:(3'reverse)AAGCGTGCCCTGGGCATCGACTCCCTTGAACAGGGACAGTCCTCTCCCCTTTCCCTGCGACGGTGGTGG;shMBP3:GATGCCCAGGGCACGCTTTCCAAAATTTTTAAGCTGGGAGGACGTGATAGTCGCTCTGGATCTCCGATG;shMBP4:(3'reverse)ACGGGCATGATCCATCGTACTTGCTGTGGCCAGGTACTTGGATCTAGAAGCCATCGGAGATCCAGAGCG;shMBP5:ACGATGGATCATGCCCGTCATGGCTTCCTCCCACGTCACCGCGACACGGGCATCCTTGACTCCATCGGG;shMBP6:(3'reverse)**AAGCTT**TCAACTAGTCCCGATGGAGTCAAGGATGCC. The NheI site in shMBP1a, the XbaI site in shMBP4, and the SpeI site in shMBP6 are underlined. The BamHI site in shMBP1a and shMBP1 is double-underlined. The EcoRI site in shMBP1a and the HindIII site in shMBP6 are bolded.

Synthetic oligonucleotides for generation and amplification of **shOSP/MS** DNA: shOSP1a:**GAATTC**GCTAGCAAGCTGGATGAGCTGGGCTCC;shOSP1:AAGCTGGATGAGCTGGGCTCCAAGGGGCTGTGGGCCGACAGCGTCATGGCAACGGGGCTG;shOSP2:(3’reverse)AACAGTCAGCAGGATGAGGATGTCCACCAGTGGCTTGCTGTGGTACAGCCCCGTTGCCATGAC;shOSP3:ATCCTGCTGCTGACTGTTCTTCCGTCTATCCGTATGGGCCAGAACCGTTTCTACTACACTGCGGGC;shOSP4:(3’reverse)CACGATGACATGCATTACGTGGGCACTCTTCGCATGAGTCGGGGAGCTAGAGCCCGCAGTGTAGTAGAA;shOSP5:GTAATGCATGTCATCGTGACCACCTCCACCAATGACTGGGTGGTGACCAGCCTGGCTCTCAGCGCC;shOSP6:(3’reverse)**AAGCTT**TCACTGCAGGTGGGCGCTCACTGGGAACCAGATGGTGGCAACAAGGGCGCTGAGAGCCAGGCT;shOSP6a:(3'reverse)**AAGCTT**TCACTGCAGGTGGGC. The NHeI site in shOSPIa, the NsiI site in shOSP4, and the PstI site in shOSP6 and shOSP6a are underlined. The SpeI site in shOSP1a is double-underlined. The EcoRI site in shOSP1a and the HindIII site in shOSP6 and shOSP6a are bolded.

Synthetic oligonucleotides for generation and amplification of **shMOBP/MS** DNA: shMOBP1a:**GAATTC**GCTAGCCTGCAGCAG;shMOBP1:**GAATTC**GCTAGCCTGCAGCAGAAGTATTCCGAACACTTCAGCATACACAGCAGCCCACCGTTCACC;shMOBP2:(3'reverse)GGTCTTCTGGCTGGCAGAGCTGATCCAGTCCTCCTCTTTATTGAGGAAGGTGAACGGTGGGCTGCT;shMOBP3:TCTGCCAGCCAGAAGACCCGTACCAGCCGCCGTGCCAAGTCCCCACAGCGTCCGAAGCAACAGCCA;shMOBP4:(3'reverse)GCTGCTGCGCGGCTGTTGCTTGTCGACGACCACCGCTGGCGGCGCAGCTGGCTGTTGCTTCGGACG;shMOBP5:CAACAGCCGCGCAGCAGCCCGCTCCGTGGGCCAGGTGCCAGCCGTGGGCTCGAGTGAAAGCTT;shMOBP6:(3'reverse)**AAGCTT**TCACTCGAG CCCACGGCT. The NHeI site in shMOBPIa and shMOBP1, the SalI site in shMOBP4, and the XhoI site in shMOBP6 are underlined. The PstI site in shMOBP1a and shMOBP1 are double-underlined. The EcoRI site in shMOBP1a and shMOBP1 and the HindIII site in shMOBP6 are bolded.

Synthetic oligonucleotides for generation and amplification of **shPLP/MS** DNA: shPLP1a:**GAATTC**GCTAGCCTCGAGTAC;shPLP1:**GAATTC**GCTAGCCTCGAGTACAAGACCACCATCAGCGGCAAGGGCCTGAGCGCAACGGTAACAGGGGGC;shPLP2:(3'reverse)ACGCTCCAAAGAATGAGCTTGATGTTGGCCTCTGGAACCACGCCCCTTCTGGCCCCCTGTTACCGTTGC;shPLP3:GCTCATTCTTTGGAGCGTGTGAGCCATTCTTTGGGAAAATGGTTAGGACATCCGGACAAGTTCAAC;shPLP4:(3'reverse)GGCAGAGGTCTTGCTTGGGAAGGCAATAGACTGGCTGGTGGTCCAGGTGTTGAACTTGTCCGGATG;shPLP5:CCAAGCAAGACCTCTGCCAGTATAGGCAGTCTCTCTGCTGACGCCGTTTCTGGCTCCAACCTTCTGTCC;shPLP6:(3'reverse)TCCAATAAACAGGTGGAAGGTCATTTGGAACTCTGCTGTTTTGCTGATGGACAGAAGGTTGGAGCC;shPLP7:TTCCACCTGTTTATTGGATCCGCCCTCACTGGCACAGAAAAGCTGATTGAGACCTATTTCTCCAAATTT;shPLP8:(3'reverse)**AAGCTT**TCATGATCAGAACTTGGTGCCACGGCCCATGAGTTTAAGGACGGCAAATTTGGAGAAATAGGT;shPLP8a:(3'reverse)**AAGCTT**TCATGATCAGAACTT. The NHeI site in shPLPIa and shPLP1, the BamHI site in shPLP7, and the BclI site in shPLP8 and shPLP8a are underlined. The XhoI site in shPLP1a and shPLP1 is double-underlined. The EcoRI site in shPLP1a and shPLP1 and the HindIII site in shPLP8a and shPLP8 are bolded.

***Construction and cloning of shMOG/MS, shMBP/MS, shOSP/MS, shMOBP/MS, and shPLP/MS***. For the preparation of DNA template of each synthetic human autoantigen gene, the relevant oligonucleotides were mixed (each 75 pmoles) in Taq DNA polymerase buffer (40 μl final volume) containing dNTPs (Cat. No RO181, MBI Fermentas, Vilnius, Lithuania) at a final concentration of 0.2 mM each, and a mixture of Vent DNA polymerase (0.2 U; cat. No 2545, New England Biolabs, MA, USA) and Taq DNA polymerase (0.5 U; cat. No AB-0192, Advanced Biotechnologies, Surrey, UK). After denaturation (94^o^C, 1 min.) and annealing (55^o^C, 2 min.), PCR overlap extension was carried out (72^o^C, 5 min.) and the resulting template (4  μl) was PCR amplified (standard conditions, 30 cycles), using the relevant oligonucleotides as primers. The shMOG/MS DNA template was generated using oligonucleotides shMOG1, 2, 3, 4, 5, and 6 and PCR amplified with oligonucleotides shMOG1a and 6a, as forward and reverse primers, respectively. The shMBP/MS DNA template was generated using oligonucleotides shMBP1, 2, 3, 4, 5, and 6 and PCR amplified with oligonucleotides shMBP1a and 6, as forward and reverse primers, respectively. The shOSP/MS DNA template was generated using oligonucleotides shOSP1, 2, 3, 4, 5, and 6 and PCR amplified with oligonucleotides shOSP1a and 6a, as forward and reverse primers, respectively. The shMOBP/MS DNA template was generated using oligonucleotides shMOBP1, 2, 3, 4, 5, and 6 and PCR amplified with oligonucleotides shMOBP1a and 6, as forward and reverse primers, respectively. The shPLP/MS DNA template was generated using oligonucleotides shPLP1, 2, 3, 4, 5, 6, 7, and 8, and PCR amplified with oligonucleotides shPLP1a and 8a, as forward and reverse primers, respectively. The amplified PCR products of the expected size eluted from agarose gel were directly cloned into a T vector (pGEM-T; cat. No A3600, Promega, Madison, WI, USA) and sequenced using sp6 and T7 specific primers.

The synthetic human autoantigen genes were also each cloned into the bacterial expression vector pRSET (Cat. No V351-20, Invitrogen, San Diego, CA, USA) to ascertain their expressibility, as described previously [40].

**Construction and expression of the Y-MSPc-encoding synthetic gene***.* To construct the Y-MSPc-encoding gene, the pRSET/shMOG/MS was linearized by cleavage at the BamHI and HindIII sites, and the DNAs of the other synthetic human autoantigen genes were cleaved out of the pRSET vector as follows: shMBP/MS DNA was cleaved out at the BamHI and SpeI sites; shOSP/MS DNA was cleaved out at the NheI and PstI sites; shMOBP/MS DNA was cleaved out at the PstI and XhoI sites; and shPLP/MS DNA was cleaved out at the XhoI and HindIII sites. The DNA fragments of the right sizes were gel-eluted, cleaned, and sequentially ligated to link shMBP/MS DNA to pRSET/shMOG/MS DNA via their BamHI sites, the shOSP/MS DNA to the shMBP/MS DNA via their SpeI/NheI sites, the shMOBP/MS DNA to the shOSP/MS DNA via their PstI sites, and the shPLP/MS DNA to the shMOBP/MS DNA via their XhoI sites and to the pRSET/shMOG/MS via their HindIII sites, as depicted in Figure 1A, to generate the pRSET/Y-MSPc expression vector. DNA sequence analysis was performed using the pRSET-specific primers to confirm the DNA sequence encoding shMOG/MS, shMBP/MS, shOSP/MS, shMOBP/MS, and shPLP/MS as an open reading frame with the ATG of the pRSET expression vector. Expression of the Y-MSPc-encoding gene cloned into pRSET and isolation of its product, Y-MSPc, by metal chelate affinity chromatography on Ni-NTA agarose were as described previously [40]. Briefly, the pRSET containing the Y-MSPc gene was transformed into *Escherichia coli* host (BL21-DE3) and protein expression was induced by isopropyl β-D-thiogalactopyranoside (IPTG; cat. No RO392, MBI Fermentas, Vilnius, Lithuania). The expressed protein was isolated under denaturing conditions (8 M urea) by metal chelate affinity chromatography on Ni-NTA agarose (Cat. No 30230, Qiagen, Chatsworth, CA) according to the manufacturer's protocol. On SDS-PAGE, the Ni-NTA-isolated protein showed (Fig. S1, lane 4) a band of about 60kd (about 70% of total protein) and two smaller bands of about 45 and 40kd (each about 10-15% of total protein). All the bands were immunoreactive with anti-MOG, anti-MBP, and anti-MOBP antibodies in western blot analysis (data not shown), suggesting that the smaller and fainter bands resulted from pre-mature termination of translation of the 60kd Y-MSPc. For further purification, the fractions obtained from the metal chelate affinity chromatography (in 8M urea) were treated with β-mercaptoethanol, dialyzed against 2M urea in Tris-HCL pH 8.0; 150mM NaCl, and subjected to high flow gel filtration on Superdex 75 Prep Grade Column 35/600 (GE Healthcare), and the major fraction (60kd Y-MSPc; Fig. S1, lane 5) was gradually dialyzed against decreasing concentrations of urea (2 M to 0 M).

In the experiments shown in this study, the Ni-NTA-isolated Y-MSPc preparation was used due to the high consumption of Y-MSPc in this study, and the relatively low yield of Superdex 75-purified 60kd YMSPc. However, the Ni-NTA-isolated Y-MSPc and the Superdex 75-purified YMSPc preparations were equally immunofunctional and effective in the suppression of PLP-induced EAE and in treatment of ongoing PLP-induced EAE in (C57Bl x SJL/J)F1 mice (data not shown).

REFERENCES

1. Kerlero de Rosbo N, Hoffman M, Mendel I, Yust I, Kaye J, et al. (1997) Predominance of the autoimmune response to myelin oligodendrocyte glycoprotein (MOG) in multiple sclerosis: reactivity to the extracellular domain of MOG is directed against three main regions. Eur J Immunol 27: 3059-3069.

2. Lindert RB, Haase CG, Brehm U, Linington C, Wekerle H, et al. (1999) Multiple sclerosis: B- and T-cell responses to the extracellular domain of the myelin oligodendrocyte glycoprotein. Brain 122 ( Pt 11): 2089-2100.

3. Kerlero de Rosbo N, Brok HP, Bauer J, Kaye JF, t Hart BA, et al. (2000) Rhesus monkeys are highly susceptible to experimental autoimmune encephalomyelitis induced by myelin oligodendrocyte glycoprotein: characterisation of immunodominant T- and B-cell epitopes. J Neuroimmunol 110: 83-96.

4. Wallstrom E, Khademi M, Andersson M, Weissert R, Linington C, et al. (1998) Increased reactivity to myelin oligodendrocyte glycoprotein peptides and epitope mapping in HLA DR2(15)+ multiple sclerosis. Eur J Immunol 28: 3329-3335.

5. Brok HP, Boven L, van Meurs M, Kerlero de Rosbo N, Celebi-Paul L, et al. (2007) The human CMV-UL86 peptide 981-1003 shares a crossreactive T-cell epitope with the encephalitogenic MOG peptide 34-56, but lacks the capacity to induce EAE in rhesus monkeys. J Neuroimmunol 182: 135-152.

6. Mendel Kerlero de Rosbo N, Ben-Nun A (1996) Delineation of the minimal encephalitogenic epitope within the immunodominant region of myelin oligodendrocyte glycoprotein: diverse V beta gene usage by T cells recognizing the core epitope encephalitogenic for T cell receptor V beta b and T cell receptor V beta a H-2b mice. Eur J Immunol 26: 2470-2479.

7. Mendel I, Kerlero de Rosbo N, Ben-Nun A (1995) A myelin oligodendrocyte glycoprotein peptide induces typical chronic experimental autoimmune encephalomyelitis in H-2b mice: fine specificity and T cell receptor V beta expression of encephalitogenic T cells. Eur J Immunol 25: 1951-1959.

8. Kerlero de Rosbo N, Mendel I, Ben-Nun A (1995) Chronic relapsing experimental autoimmune encephalomyelitis with a delayed onset and an atypical clinical course, induced in PL/J mice by myelin oligodendrocyte glycoprotein (MOG)-derived peptide: preliminary analysis of MOG T cell epitopes. Eur J Immunol 25: 985-993.

9. Johns TG, Kerlero de Rosbo N, Menon KK, Abo S, Gonzales MF, et al. (1995) Myelin oligodendrocyte glycoprotein induces a demyelinating encephalomyelitis resembling multiple sclerosis. J Immunol 154: 5536-5541.

10. Rich C, Link JM, Zamora A, Jacobsen H, Meza-Romero R, et al. (2004) Myelin oligodendrocyte glycoprotein-35-55 peptide induces severe chronic experimental autoimmune encephalomyelitis in HLA-DR2-transgenic mice. Eur J Immunol 34: 1251-1261.

11. Amor S, Groome N, Linington C, Morris MM, Dornmair K, et al. (1994) Identification of epitopes of myelin oligodendrocyte glycoprotein for the induction of experimental allergic encephalomyelitis in SJL and Biozzi AB/H mice. J Immunol 153: 4349-4356.

12. Yu M, Kinkel RP, Weinstock-Guttman B, Cook DJ, Tuohy VK (1998) HLA-DP: a class II restriction molecule involved in epitope spreading during the development of multiple sclerosis. Hum Immunol 59: 15-24.

13. Pelfrey CM, Trotter JL, Tranquill LR, McFarland HF (1993) Identification of a novel T cell epitope of human proteolipid protein (residues 40-60) recognized by proliferative and cytolytic CD4+ T cells from multiple sclerosis patients. J Neuroimmunol 46: 33-42.

14. Greer JM, Sobel RA, Sette A, Southwood S, Lees MB, et al. (1996) Immunogenic and encephalitogenic epitope clusters of myelin proteolipid protein. J Immunol 156: 371-379.

15. Ohashi T, Yamamura T, Inobe J, Kondo T, Kunishita T, et al. (1995) Analysis of proteolipid protein (PLP)-specific T cells in multiple sclerosis: identification of PLP 95-116 as an HLA-DR2,w15-associated determinant. Int Immunol 7: 1771-1778.

16. Trotter JL, Pelfrey CM, Trotter AL, Selvidge JA, Gushleff KC, et al. (1998) T cell recognition of myelin proteolipid protein and myelin proteolipid protein peptides in the peripheral blood of multiple sclerosis and control subjects. J Neuroimmunol 84: 172-178.

17. Greer JM, Csurhes PA, Cameron KD, McCombe PA, Good MF, et al. (1997) Increased immunoreactivity to two overlapping peptides of myelin proteolipid protein in multiple sclerosis. Brain 120 ( Pt 8): 1447-1460.

18. Tuohy VK, Sobel RA, Lu Z, Laursen RA, Lees MB (1992) Myelin proteolipid protein: minimum sequence requirements for active induction of autoimmune encephalomyelitis in SWR/J and SJL/J mice. J Neuroimmunol 39: 67-74.

19. Mangalam AK, Khare M, Krco C, Rodriguez M, David C (2004) Identification of T cell epitopes on human proteolipid protein and induction of experimental autoimmune encephalomyelitis in HLA class II-transgenic mice. Eur J Immunol 34: 280-290.

20. Tuohy VK (1994) Peptide determinants of myelin proteolipid protein (PLP) in autoimmune demyelinating disease: a review. Neurochem Res 19: 935-944.

21. Pelfrey CM, Trotter JL, Tranquill LR, McFarland HF (1994) Identification of a second T cell epitope of human proteolipid protein (residues 89-106) recognized by proliferative and cytolytic CD4+ T cells from multiple sclerosis patients. J Neuroimmunol 53: 153-161.

22. Endoh M, Kunishita T, Nihei J, Nishizawa M, Tabira T (1990) Susceptibility to proteolipid apoprotein and its encephalitogenic determinants in mice. Int Arch Allergy Appl Immunol 92: 433-438.

23. Sireci G, Dieli F, Caccamo N, Barera A, Carta P, et al. (2003) A human leucocyte antigen-DR1 transgene confers susceptibility to experimental allergic encephalomyelitis elicited by an epitope of myelin basic protein. Scand J Immunol 58: 188-194.

24. Fritz RB, Skeen MJ, Chou CH, Zamvil SS (1990) Localization of an encephalitogenic epitope for the SJL mouse in the N-terminal region of myelin basic protein. J Neuroimmunol 26: 239-243.

25. Offner H, Hashim GA, Celnik B, Galang A, Li XB, et al. (1989) T cell determinants of myelin basic protein include a unique encephalitogenic I-E-restricted epitope for Lewis rats. J Exp Med 170: 355-367.

26. Sakai K, Sinha AA, Mitchell DJ, Zamvil SS, Rothbard JB, et al. (1988) Involvement of distinct murine T-cell receptors in the autoimmune encephalitogenic response to nested epitopes of myelin basic protein. Proc Natl Acad Sci U S A 85: 8608-8612.

27. Ota K, Matsui M, Milford EL, Mackin GA, Weiner HL, et al. (1990) T-cell recognition of an immunodominant myelin basic protein epitope in multiple sclerosis. Nature 346: 183-187.

28. Pette M, Fujita K, Wilkinson D, Altmann DM, Trowsdale J, et al. (1990) Myelin autoreactivity in multiple sclerosis: recognition of myelin basic protein in the context of HLA-DR2 products by T lymphocytes of multiple-sclerosis patients and healthy donors. Proc Natl Acad Sci U S A 87: 7968-7972.

29. Ristori G, Giubilei F, Giunti D, Perna A, Gasperini C, et al. (2000) Myelin basic protein intramolecular spreading without disease progression in a patient with multiple sclerosis. J Neuroimmunol 110: 240-243.

30. Vergelli M, Kalbus M, Rojo SC, Hemmer B, Kalbacher H, et al. (1997) T cell response to myelin basic protein in the context of the multiple sclerosis-associated HLA-DR15 haplotype: peptide binding, immunodominance and effector functions of T cells. J Neuroimmunol 77: 195-203.

31. Das P, Drescher KM, Geluk A, Bradley DS, Rodriguez M, et al. (2000) Complementation between specific HLA-DR and HLA-DQ genes in transgenic mice determines susceptibility to experimental autoimmune encephalomyelitis. Hum Immunol 61: 279-289.

32. Madsen LS, Andersson EC, Jansson L, krogsgaard M, Andersen CB, et al. (1999) A humanized model for multiple sclerosis using HLA-DR2 and a human T-cell receptor. Nat Genet 23: 343-347.

33. Tabira T, Kira JI.(1992) In Myelin:Biology and Chemistry. RE Martenson, ed. CRC Press, USA; , p. 783-99.

34. Vu T, Myers LW, Ellison GW, Mendoza F, Bronstein JM (2001) T-cell responses to oligodendrocyte-specific protein in multiple sclerosis. J Neurosci Res 66: 506-509.

35. de Rosbo NK, Kaye JF, Eisenstein M, Mendel I, Hoeftberger R, et al. (2004) The myelin-associated oligodendrocytic basic protein region MOBP15-36 encompasses the immunodominant major encephalitogenic epitope(s) for SJL/J mice and predicted epitope(s) for multiple sclerosis-associated HLA-DRB1*1501. J Immunol 173: 1426-1435.

36. Kaushansky N, Altmann DM, Ascough S, David CS, Lassmann H, et al. (2009) HLA-DQB1*0602 determines disease susceptibility in a new "humanized" multiple sclerosis model in HLA-DR15 (DRB1*1501;DQB1*0602) transgenic mice. J Immunol 183: 3531-3541.

37. Kaye JF, Kerlero de Rosbo N, Mendel I, Flechter S, Hoffman M, et al. (2000) The central nervous system-specific myelin oligodendrocytic basic protein (MOBP) is encephalitogenic and a potential target antigen in multiple sclerosis (MS). J Neuroimmunol 102: 189-198.

38. Ben-Nun A, Kerlero de Rosbo N, Kaushansky N, Eisenstein M, Cohen L, et al. (2006) Anatomy of T cell autoimmunity to myelin oligodendrocyte glycoprotein (MOG): prime role of MOG44F in selection and control of MOG-reactive T cells in H-2b mice. Eur J Immunol 36: 478-493.

39. Kaushansky N, Hemo R, Eisenstein M, Ben-Nun A (2007) OSP/claudin-11-induced EAE in mice is mediated by pathogenic T cells primarily governed by OSP192Y residue of major encephalitogenic region OSP179-207. Eur J Immunol 37: 2018-2031.

40. Buus S, Lauemoller SL, Worning P, Kesmir C, Frimurer T, et al. (2003) Sensitive quantitative predictions of peptide-MHC binding by a 'Query by Committee' artificial neural network approach. Tissue Antigens 62: 378-384.

41. Zhong MC, Kerlero de Rosbo N, Ben-Nun A (2002) Multiantigen/multiepitope-directed immune-specific suppression of "complex autoimmune encephalomyelitis" by a novel protein product of a synthetic gene. J Clin Invest 110: 81-90.

42. Ben-Nun A, Lando Z (1983) Detection of autoimmune cells proliferating to myelin basic protein and selection of T cell lines that mediate experimental autoimmune encephalomyelitis (EAE) in mice. J Immunol 130: 1205-1209.

43. Zhong MC, Cohen L, Meshorer A, Kerlero de Rosbo N, Ben-Nun A (2000) T-cells specific for soluble recombinant oligodendrocyte-specific protein induce severe clinical experimental autoimmune encephalomyelitis in H-2(b) and H-2(s) mice. J Neuroimmunol 105**:** 39-45.
