# Supplementary figures and images for: ‘Multi-Epitope-Targeted’ Immune-Specific Therapy for a Multiple Sclerosis-Like Disease via Engineered Multi-Epitope Protein Is Superior to Peptides
Source: PLoS One. 2011 Nov 29;6(11):e27860. doi: 10.1371/journal.pone.0027860 (PMC3226621; doi:10.1371/journal.pone.0027860)

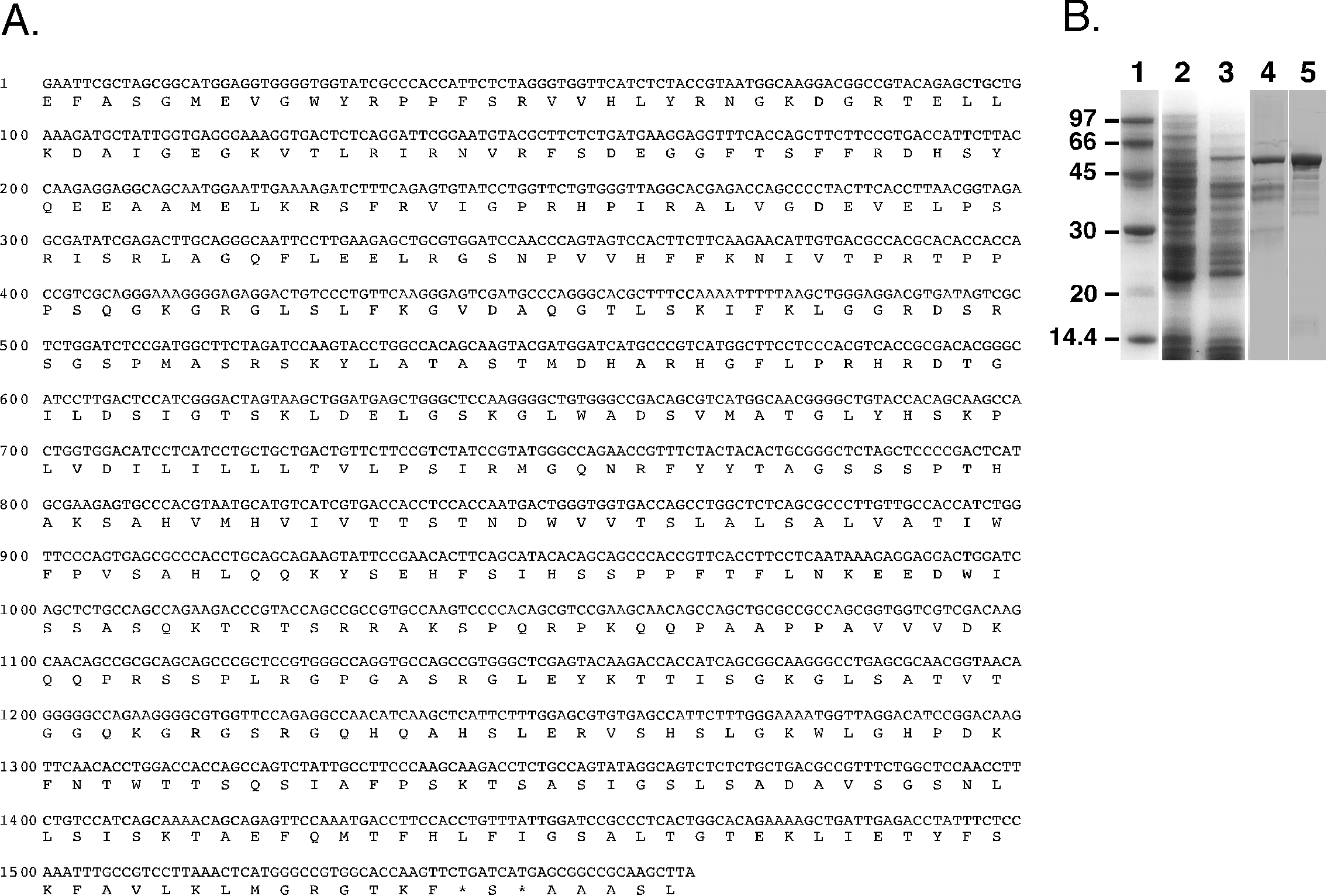

Supplement: Figure S1 — DNA sequence and expression of the Y-MSPc-encoding synthetic gene. (A) The DNA and derived amino acid sequences of Y-MSPc were confirmed and shown to be in an open reading frame with the ATG of pRSET expression vector by DNA sequence analysis using pRSET-specific primers. (B) Bacterial expression of the Y-MSPc-encoding gene and isolation of Y-MSPc. Coomassie blue-stained SDS-PAGE analysis: Lane 1, molecular weight standards; lane 2, bacterial extract before IPTG induction; lane 3, after IPTG induction; lane 4, recombinant Y-MSPc isolated by metal-chelate affinity chromatography on Ni-NTA agarose (2 µg); lane 5, Ni-NTA-isolated Y-MSPc after high flow gel filtration on Superdex 75 (60 kd fraction; 3 µg). [The smaller and fainter bands (∼50 and 40 kd ) in lane 4 were shown to result of pre-mature termination of translation of the 60 kd Y-MSPc] (TIF) [file pone.0027860.s002.tif]
