# Supplementary material for: ‘Multi-Epitope-Targeted’ Immune-Specific Therapy for a Multiple Sclerosis-Like Disease via Engineered Multi-Epitope Protein Is Superior to Peptides
Source: PLoS One. 2011 Nov 29;6(11):e27860. doi: 10.1371/journal.pone.0027860 (PMC3226621; doi:10.1371/journal.pone.0027860)
Supplement: Table S1 — The encephalitpogenic potential of (C57Bl/6JxSJL/)F1-derived T-cell lines specific for different encephalitogenic myelin epitopes. (C57Bl/6JxSJL/)F1 mice were injected s.c. with 100 µg of either peptide emulsified in CFA. T cell lines specific for the immunizing peptide were selected in-vitro from the draining LN cells that obtained 10 days after immunization, as previously described [7], [42]. Line T-cells at indicated numbers were injected i.v. into slightly irradiated (400 rads) syngeneic naïve recipients. Recipients were followed for development of clinical signs of EAE and scored as described [43]. (DOC) [file pone.0027860.s003.doc]

**Table S1.**

| Specificity of T-cell lines to myelin epitopes | Incidence | Mean day of disease onset | Mean maximal clinical score | No. of T-cells transferred |
| --- | --- | --- | --- | --- |
|  |  |  |  |  |
| phMBP89-104 | 3/3 | 7±0.6 | 4±0.5 | 2x10^6^ |
|  |  |  |  |  |
| pPLP139-151 | 5/5 | 8±1 | 5.5±0.5 | 2x10^6^ |
| pPLP175-194 | 4/4 | 13±0 | 4±0 | 5x10^6^ |
|  |  |  |  |  |
| phMOG34-56 | 5/5 | 8±0.8 | 4±0.5 | 2x10^6^ |
|  |  |  |  |  |
| phMOBP15-36 | 3/3 | 8±2 | 4±0.6 | 3x10^6^ |
| phMOBP55-77 | 4/4 | 10±1 | 2.5±0.6 | 3x10^6^ |
|  |  |  |  |  |
| phOSP55-80 | 3/3 | 9±2 | 4±0.6 | 2x10^6^ |
