# Supplementary material for: ‘Multi-Epitope-Targeted’ Immune-Specific Therapy for a Multiple Sclerosis-Like Disease via Engineered Multi-Epitope Protein Is Superior to Peptides
Source: PLoS One. 2011 Nov 29;6(11):e27860. doi: 10.1371/journal.pone.0027860 (PMC3226621; doi:10.1371/journal.pone.0027860)
Supplement: Table S2 — List of myelin peptides used in this study. (DOC) [file pone.0027860.s004.doc]

## Table S2

| Peptide | Amino acid sequence ____________________________ |
| --- | --- |
| phMOG1-22 | GQFRVIGPRHPIRALVGDEVEL |
| phMOG34-56 | GMEVGWYRPPFSRVVHLYRNGKD |
| phMOG94-116 | GGFTCFFRDHSYQEEAAMELKVE |
| phMOG202-218 | LHRRLAGQFLEELRNPF |
| phMBP11-30 | GSKYLATASTMDHARHGFLP |
| phMBP84-102 | NPVVHFFKNIVTPRTPPPS |
| phMBP89-104 | FFKNIVTPRTPPPSQG |
| phMBP147-162 | QGTLSKIFKLGGRDSR |
| phOSP22-46 | VTTSTNDWVVTCGYTIPTCRKLDEL |
| phOSP55-71 | DCVMATGLYHCKPLVDI |
| phOSP55-74 | DCVMATGLYHCKPLVDILIL |
| phOSP55-80 | DCVMATGLYHCKPLVDILILPGYVQA |
| phOSP103-123 | LPCIRMGQEPGVAKYRRAQLA |
| phOSP142-161 | PVCAHRETTIVSFGYSLYAG |
| phOSP179-201 | AGDAQAFGENRFYYTAGSSSPTH |
| phMOBP15-36 | QKYSEHFSIHCCPPFTFLNSKK |
| phMOBP55-77 | KEEDWICCACQKTRTSRRAKSPQ |
| phMOBP158-181 | QPRSSPLRGPGASRGGSPVKASRF |
| phPLP41-60 | GTEKLIETYFSKNYQDYEYL |
| phPLP139-151 | HCLGKWLGHPDKF |
| phPLP178-191 | NTWTTCQSIAFPSK |
| phPLP215-235 | PGKVCGSNLLSICKTAEFQMT |
| phPLP258-276 | IAATYNFAVLKLMGRGTKF |
